# Characterization data and kinetic studies of novel lipophilic analogues from 2,4-dichlorophenoxyacetic acid and Propanil herbicides

**DOI:** 10.1016/j.dib.2020.106202

**Published:** 2020-08-21

**Authors:** Larissa M. Porciuncula, Alex R. Teixeira, Maria F.C. Santos, Marcelo G.M. D'Oca, Elisa S. Orth, Caroline R.M. D'Oca

**Affiliations:** a Laboratorio Kolbe de Síntese Orgânica, Escola de Química e Alimentos, Universidade Federal do Rio Grande, Av. Itália, Km 08, s/n, Rio Grande, RS, Brazil; b Grupo de Catálise e Cinética, Universidade Federal do Paraná, Curitiba, PR, Brazil; c Laboratório de Ressonância Magnética Nuclear, Departamento de Química, Universidade Federal do Paraná, Curitiba, PR, Brazil

**Keywords:** Herbicides, 2, 4-D, Fatty acid amides

## Abstract

This work describes the data collection of new lipophilic esters and amides herbicides, analogues to 2*,*4*-*dichlorophenoxyacetic acid (2,4-D) and Propanil. The data include ^1^H and ^13^C NMR spectra and UV–VIS spectroscopic experiments, from the work “Novel lipophilic analogues from 2,4-D and Propanil herbicides: Biological activity and kinetic studies”. The UV–VIS and ^1^H NMR spectra were employed to kinetic degradation design, and could be used to access new herbicides derivatives with better environmental properties.

**Specifications Table**

**Table:** 

Subject	Organic chemistry
Specific subject area	Organic synthesis; Physico-Chemistry.
Type of data	Figures
How data were acquired	NMR experiments were performed on a Bruker AVANCE 400 NMR spectrometer operating at 9.4T, observing ^1^H and ^13^C at 400.13 MHz and 100.50 MHz, respectively, equipped with a 5 mm direct detection probe (BBO) with gradient along the z-axis in CDCl_3_ or DMSO‑*d6* solution with TMS as the internal standard. For qNMR ^1^H experiments, pulse was calculated by pulsecal. The relaxation delay for use in the acquisition of the quantitative ^1^H NMR spectra was determined by T1 measurements with the aid of the pulse sequence inversion recovery, with same parameters as for ^1^H spectra changing the τ values from 0.01 to 15 s. ^1^H spectra were acquired by using a 30° pulse sequence (zg) with the following parameters: 30 s of relaxation delay (D1), 16 transients, a spectral width (SW) of 4789.27 Hz (∼ 12.0 ppm), 64 K numbers of data (TD), and 6.84 s of acquisition time (AQ). The experiments were performed at 298 K. FIDs were Fourier transformed with line broadening (LB) = 0.3 Hz. The resulting spectra were manually phased and baseline corrected, and referenced to the TMS at δ 0.0 ppm. The kinetic studies were carried by UV–Vis spectroscopy (Agilent Cary). Infrared (IR) spectra were acquired on a Schimadzu IR PRESTIGIE-21.
Data format	Raw and analyzed data
Parameters for data collection	The kinetic UV–Vis spectroscopy (Agilent Cary) monitored the region of 190–800 nm under pseudo-first order conditions. An aliquot of 20 μL stock MeCN solution (0.01 mol. L^−1^) was added to a quartz cuvette (10 mm optical path) containing 3 mL of the reaction medium: acid solution (HCl 0.1 mol. L^−1^) or alkaline solution (NaOH 0.1 mol. L^−1^). The reactions were monitored for at least five half-life times, by following the reactant consumption and product formation. The kinetic profiles were fitted with equations, using iterative least-squares software.
Description of data collection	The NMR spectroscopic data were collected from isolated product, from chromatographic column. Kinetic data (UV–VIS and ^1^H NMR) were collected from aliquots directly retired from reaction, under alkaline or acid conditions.
Data source location	Universidade Federal do Paraná, Centro Politécnico, Curitiba, Paraná, Brazil.
Data accessibility	With the article
Related research article	L. M. Porciuncula, A. R. Teixeira, M. F. C Santos, M. G. M. D'Oca, L. S. Santos, F. Nachtigall, E. S. Orth, C. R. M. D'Oca, Novel lipophilic analogues from 2,4-D and Propanil herbicides: Biological activity and kinetic studies, Chem. Phys. Lipids. DOI: 10.1016/j.chemphyslip.2020.104947

**Value of the Data**

• These data are useful or important because describe the spectroscopic data of the lipophilic amides and esters analogs from classical organochlorides herbicides. In addition, the data showed the kinetic parameters obtained in acid and alkaline hydrolysis after the incorporation of fatty long-chains in herbicides.
• This dataset could be useful for other research groups interesting in the characterization of new derivatives of organochlorides herbicides and can benefit kinetic parameter studies relational to organochlorides herbicides degradation in the environmental.
• This dataset can be used for application and in the development of experiments in agricultural practices with environmental-friendly agrochemicals. Annually, around 2.5 million tons of agrochemicals are used worldwide and this causes an impact on the environment such as water suppliers and soil.

## 1 Data Description

The dataset referring to lipophilic analogues from herbicides 2,4-dichlorophenoxyacetic acid (2,4-D) and Propanil that were obtained from fatty common alkyl chains. The synthesis of new lipophilic esters **6a-c** was realized from esterification reaction of herbicide 2,4-D with palmitic (C16:0), stearic (C18:0) and oleic (C18:1) fatty alcohols. The experiments were performed according to previous work using sulfamic acid (H_2_NSO_3_H) catalyst [1]. After synthesis of the fatty esters, the synthesis of lipophilic amides **8a-c** from 2,4-D was investigated from different methodologies. The synthesis of new fatty amines **11a-c** was derived from 3,4-dichloroaniline, common core present in Propanil, Linuron and Diuron agrochemicals. The lipophilic esters and amides synthesized from 2,4-D and 3,4-dichloroaniline were characterized by ^1^H and ^13^C NMR, infrared spectroscopy. Afterwards, the lipophilic herbicides **6a-c, 8a-c** and **11a-c** were submitted to studies of kinetic behavior in aqueous medium, under basic and acid conditions. The degradation's profile was studied by kinetic UV–vis and ^1^H NMR experiments.

### 1.1 Characterization data by NMR experiments

The characterization dates of lipophilic herbicides **6a-c, 8a-c** and **11a-c** are showed in the Fig. 1, Fig. 2, Fig. 3, Fig. 4, Fig. 5, Fig. 6, Fig. 7, Fig. 8, Fig. 9, Fig. 10, Fig. 11, Fig. 12, Fig. 13, Fig. 14, Fig. 15, Fig. 16, Fig. 17, Fig. 18, Fig. 19, Fig. 20, Fig. 21, Fig. 22, Fig. 23, Fig. 24, Fig. 25, Fig. 26, Fig. 27, Fig. 28, Fig. 29, Fig. 30.

**Fig. 1:**
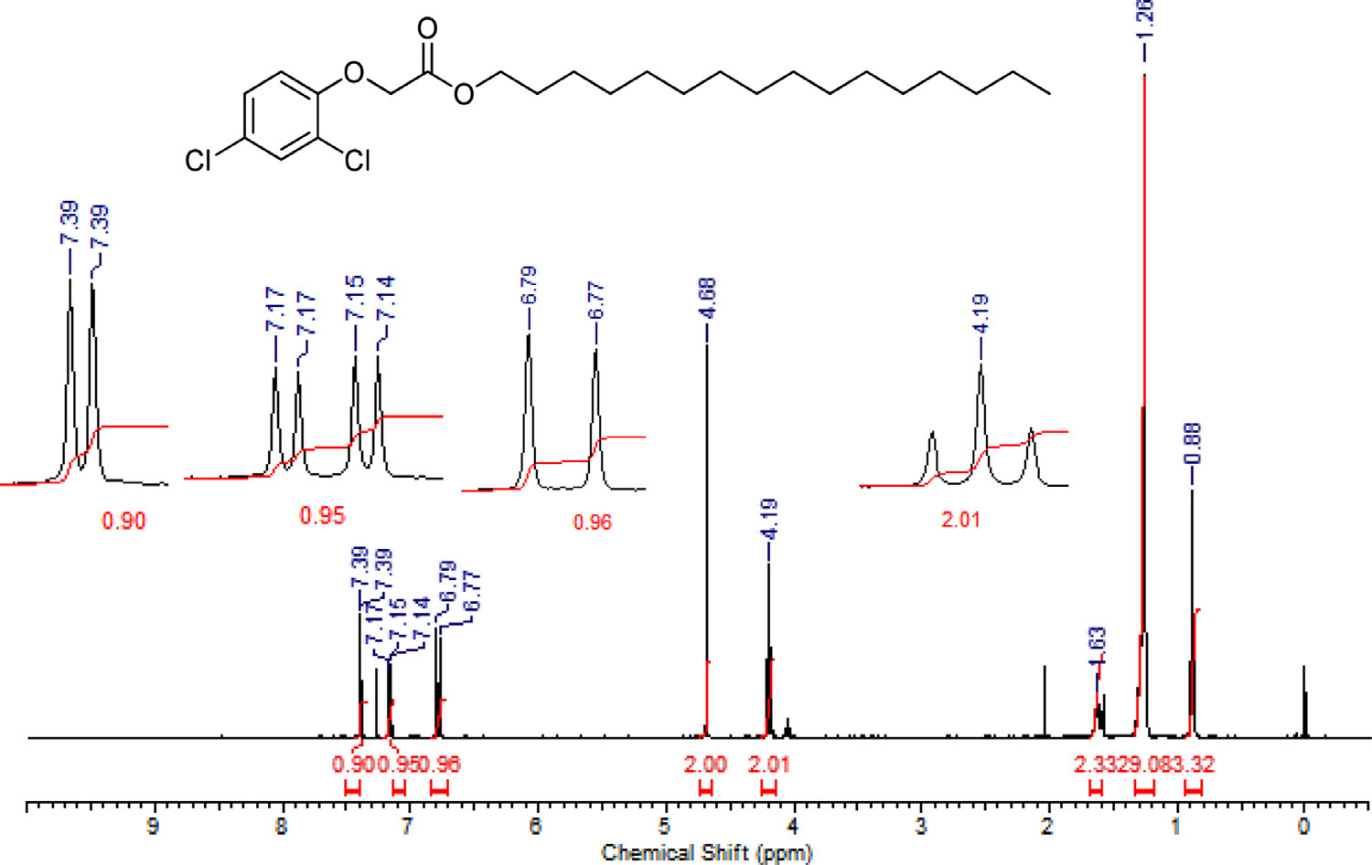
^1^H NMR (CDCl_3,_ 400 MHz) spectrum of compound **6a**.

**Fig. 2:**
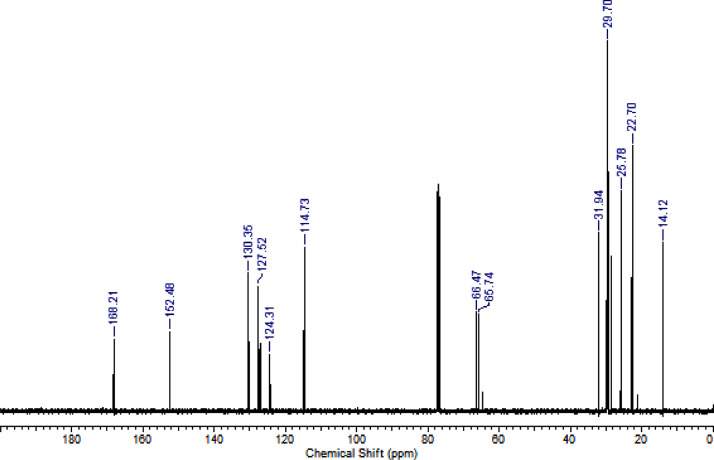
^13^C NMR (CDCl_3,_ 100 MHz) spectrum of compound **6a**.

**Fig. 3:**
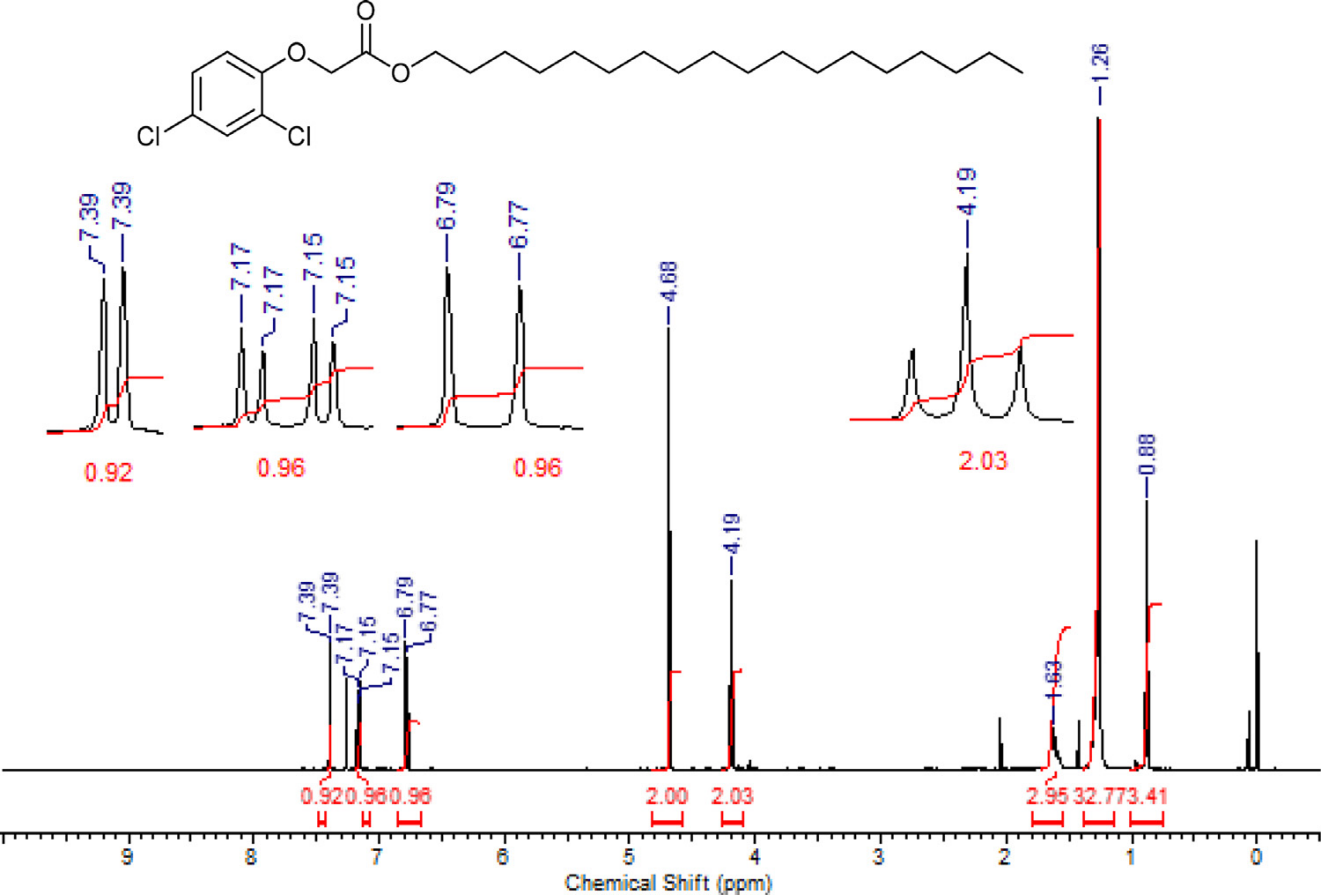
^1^H NMR (CDCl_3,_ 400 MHz) spectrum of compound **6b**.

**Fig. 4:**
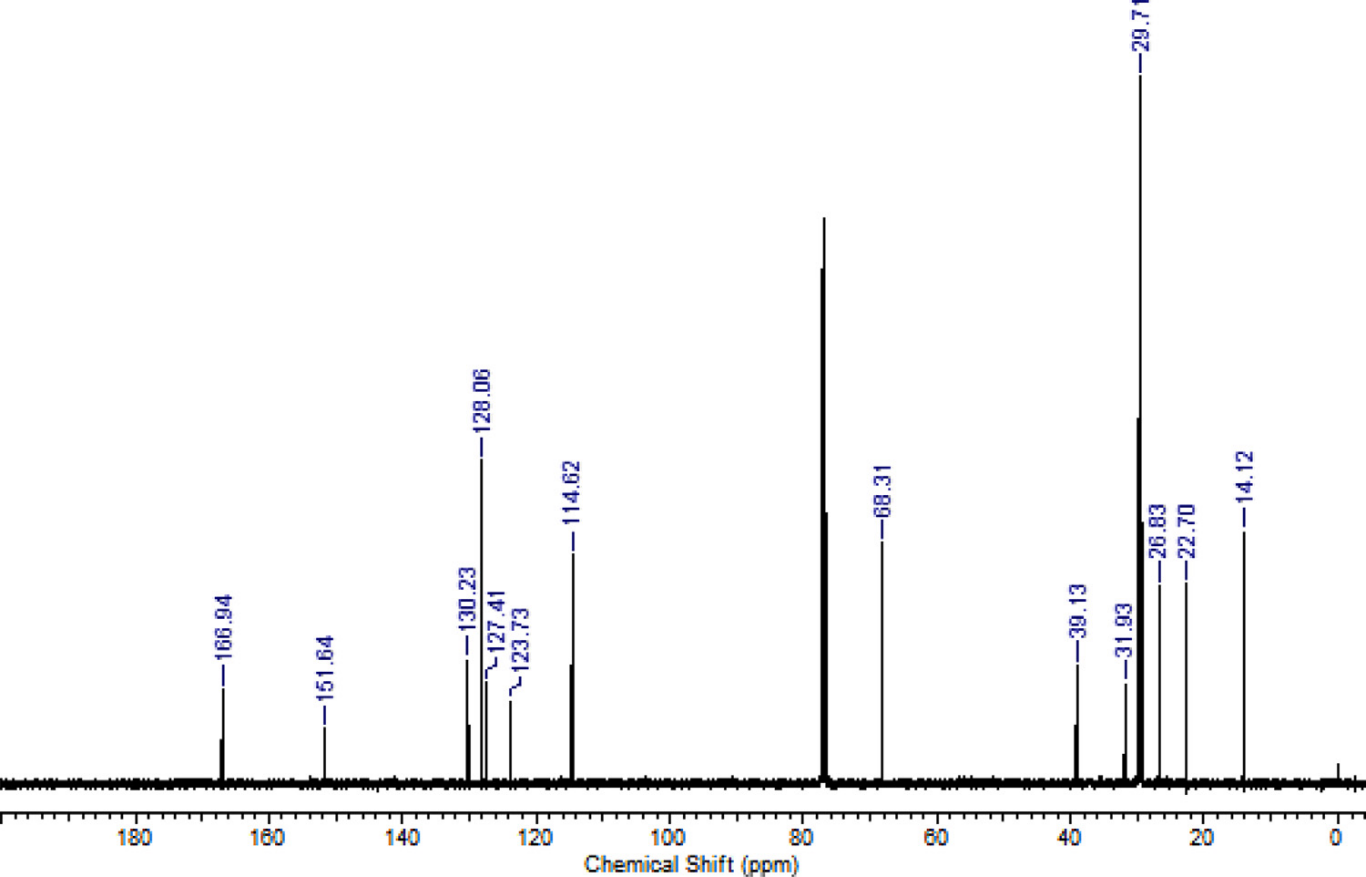
^13^C NMR (CDCl_3,_ 100 MHz) spectrum of compound **6b**.

**Fig. 5:**
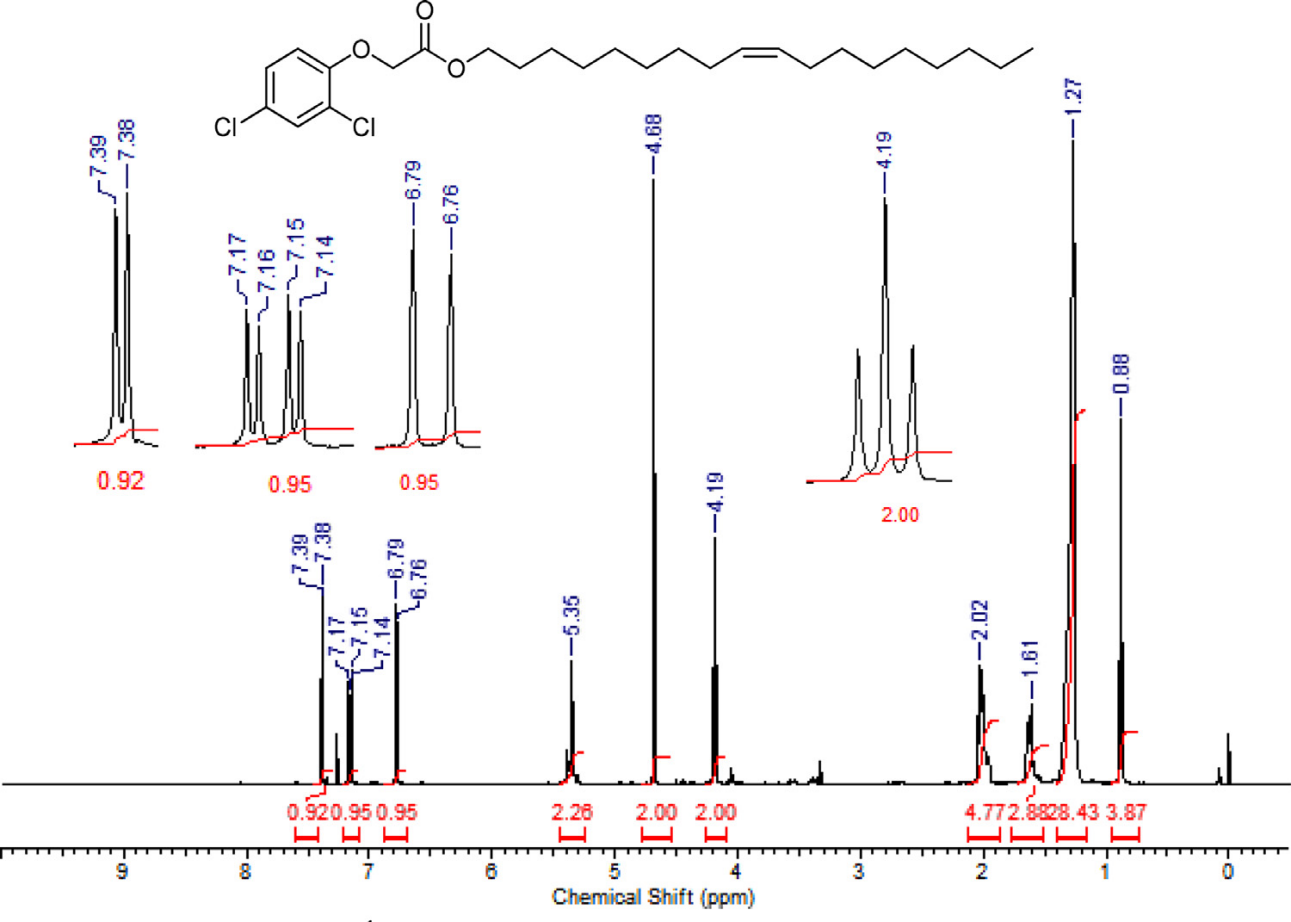
^1^H NMR (CDCl_3,_ 400 MHz) spectrum of compound **6c**.

**Fig. 6:**
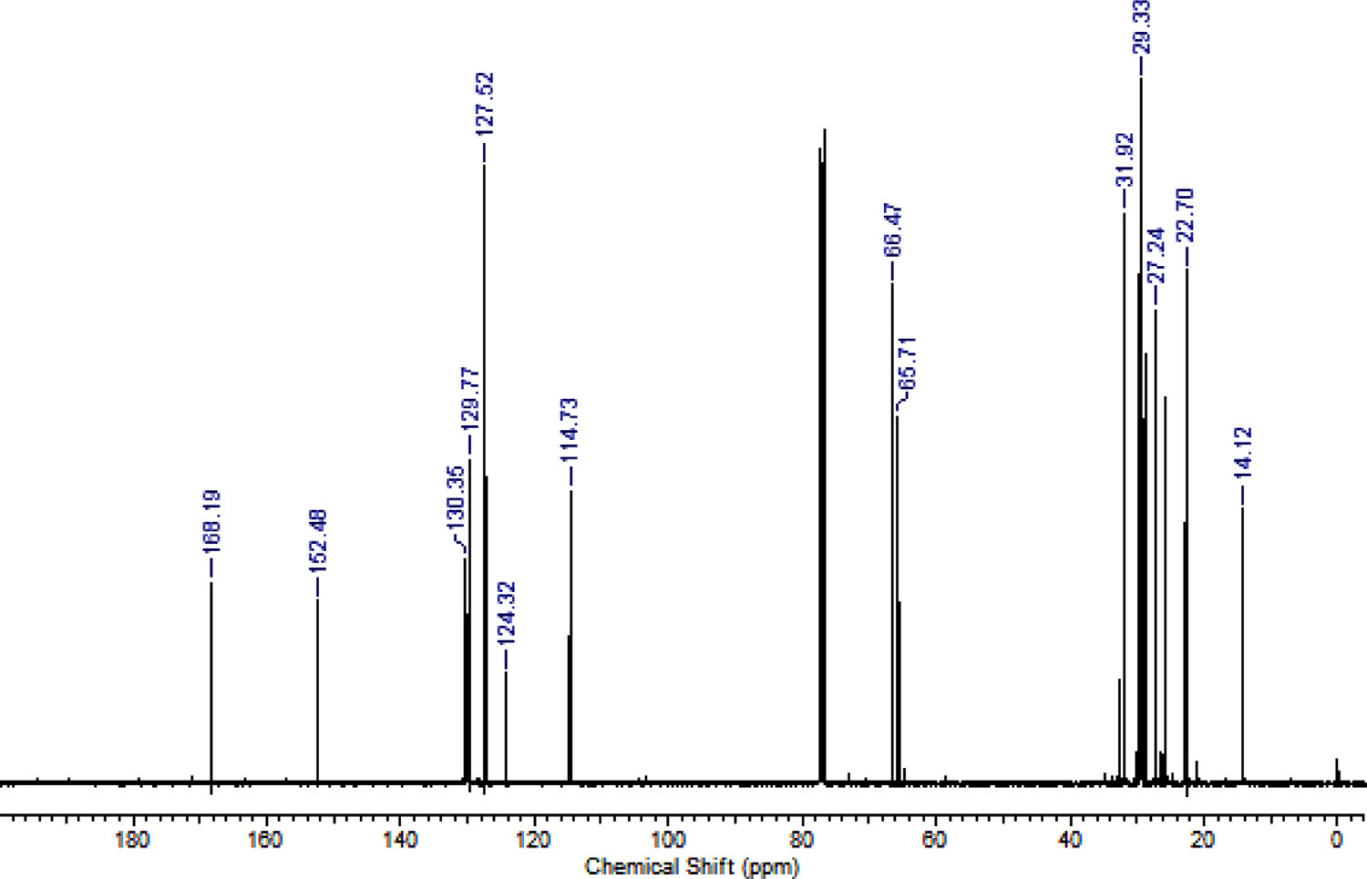
^13^C NMR (CDCl_3,_ 100 MHz) spectrum of compound **6c**.

**Fig. 7:**
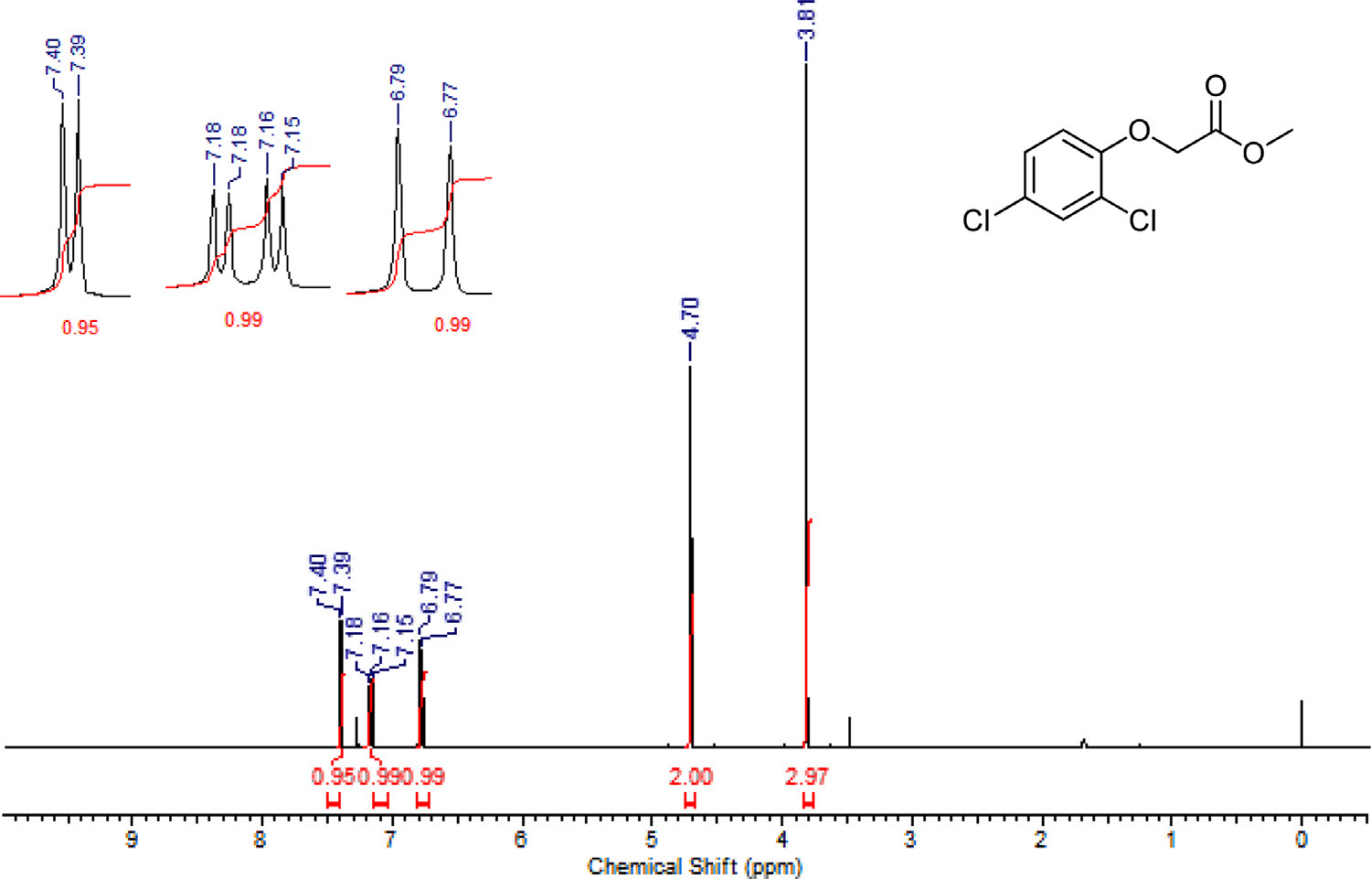
^1^H NMR (CDCl_3,_ 400 MHz) spectrum of compound **6d**

**Fig. 8:**
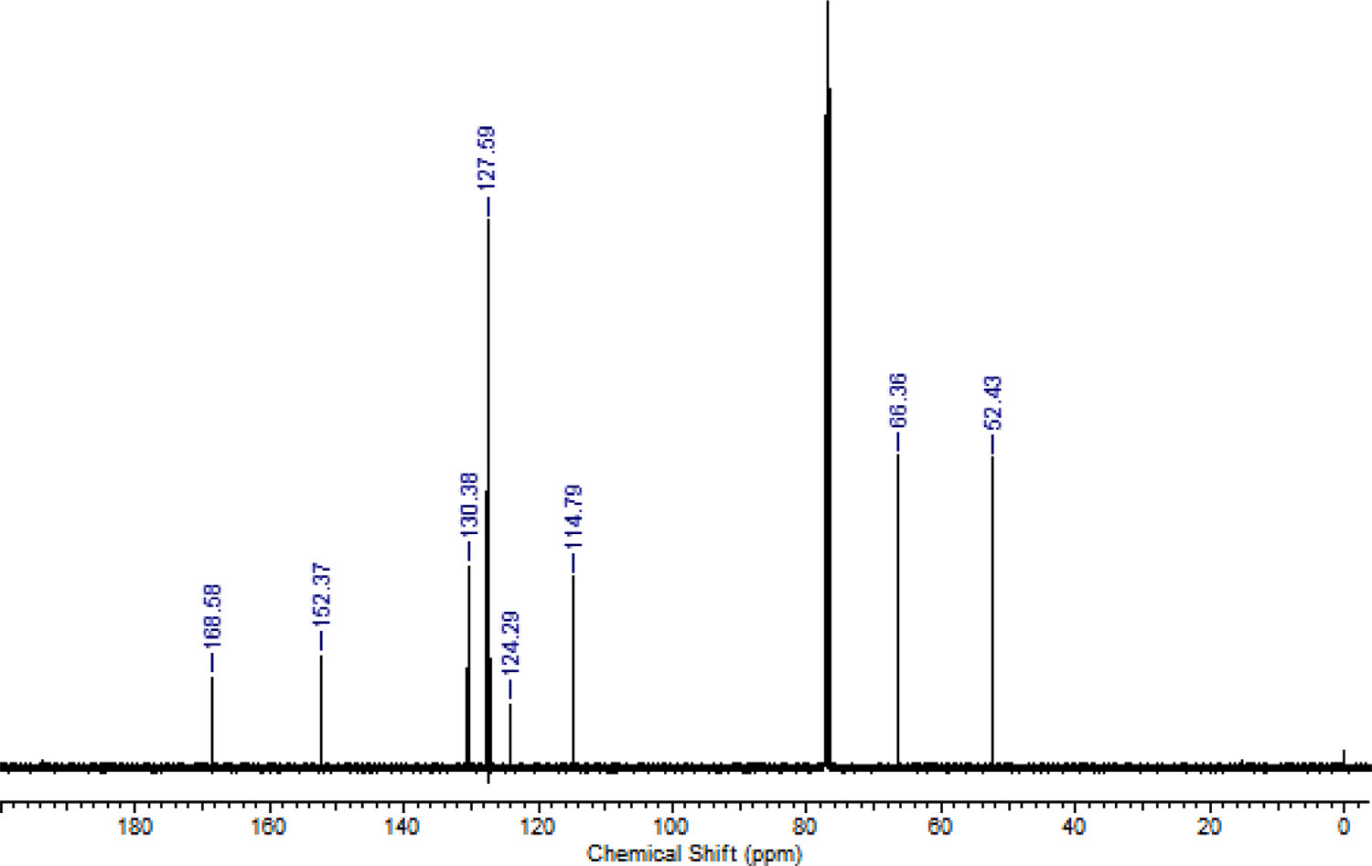
^13^C NMR (CDCl_3,_ 100 MHz) spectrum of compound **6d**

**Fig. 9:**
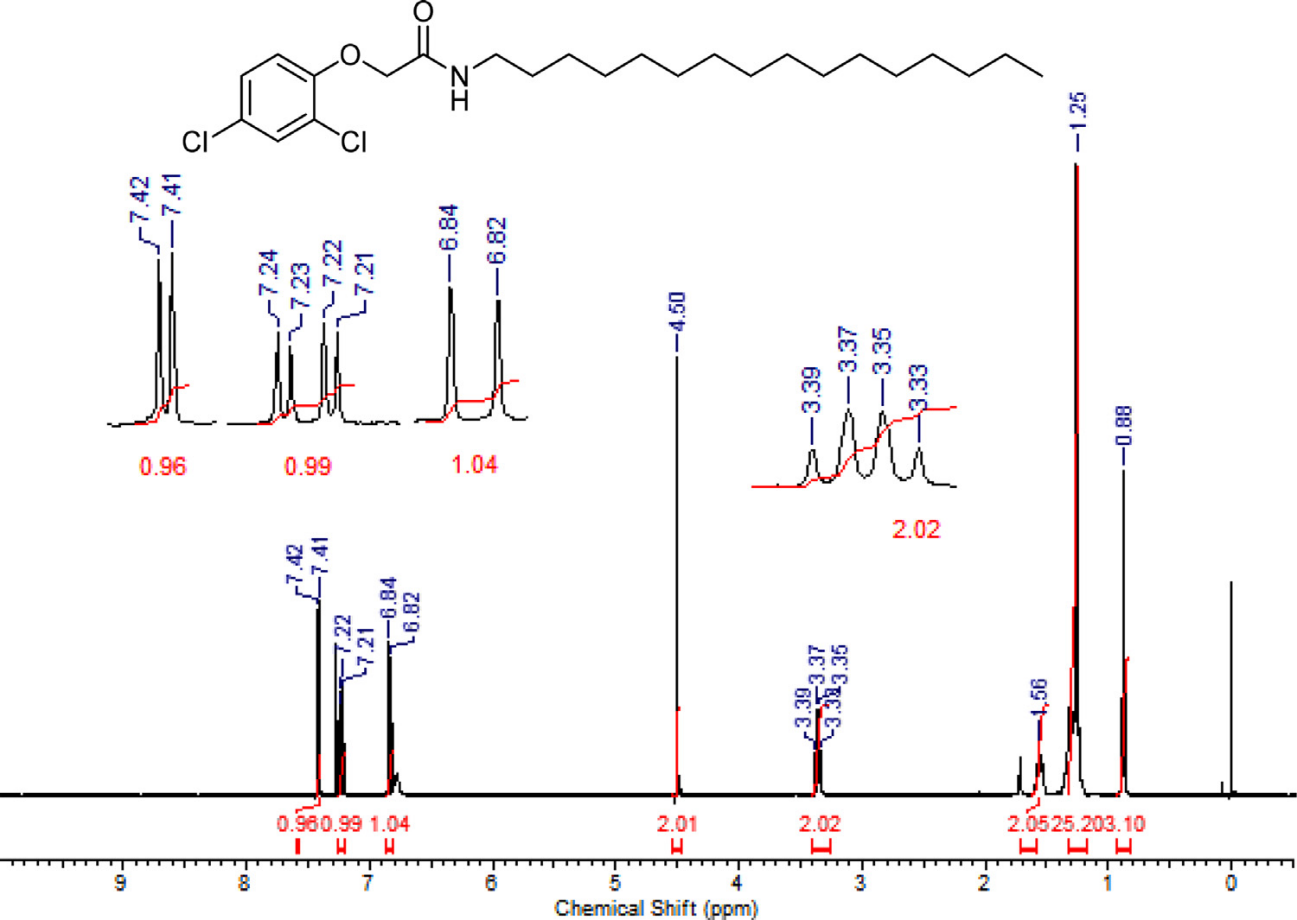
^1^H NMR (CDCl_3,_ 400 MHz) spectrum of compound **8a**.

**Fig. 10:**
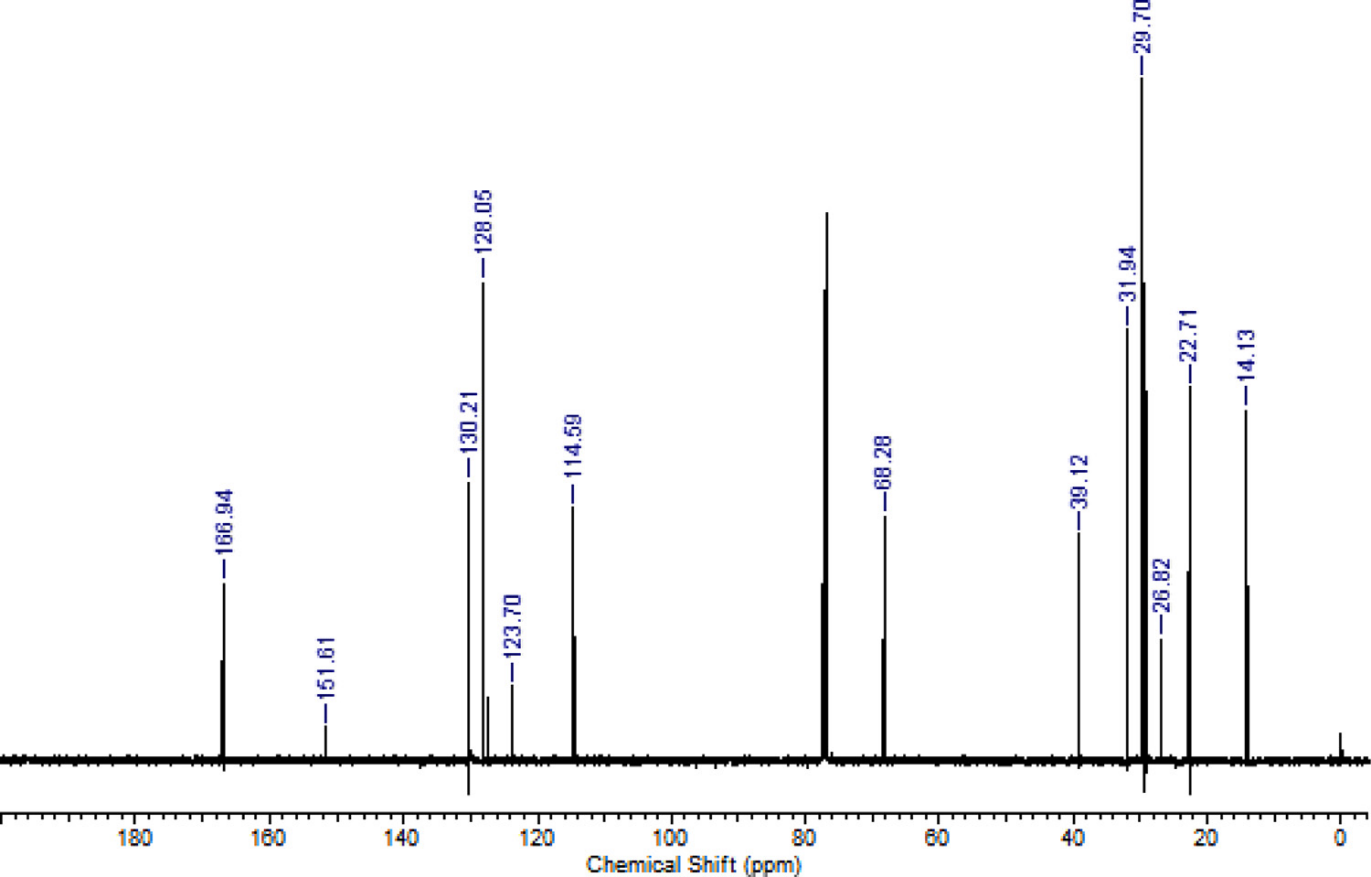
^13^C NMR (CDCl_3,_ 100 MHz) spectrum of compound **8a**.

**Fig. 11:**
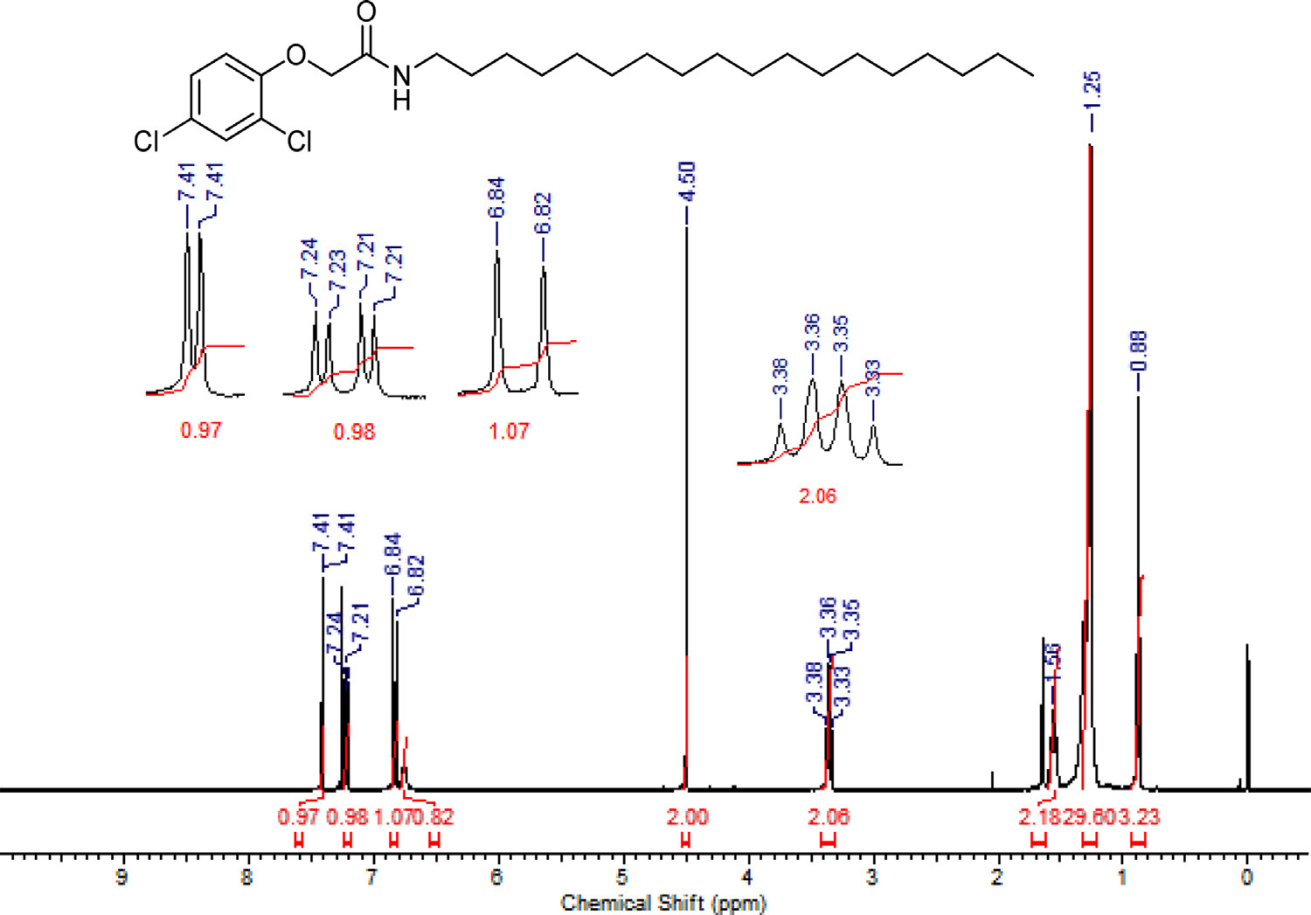
^1^H NMR (CDCl_3,_ 400 MHz) spectrum of compound **8b**.

**Fig. 12:**
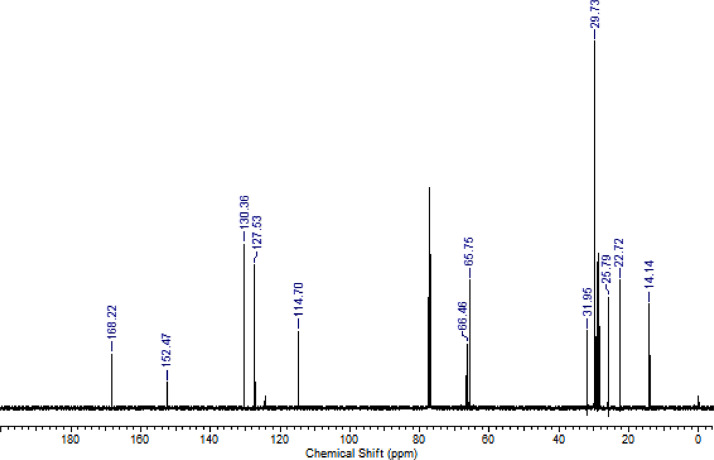
^13^C NMR (CDCl_3,_ 100 MHz) spectrum of compound **8b**.

**Fig. 13:**
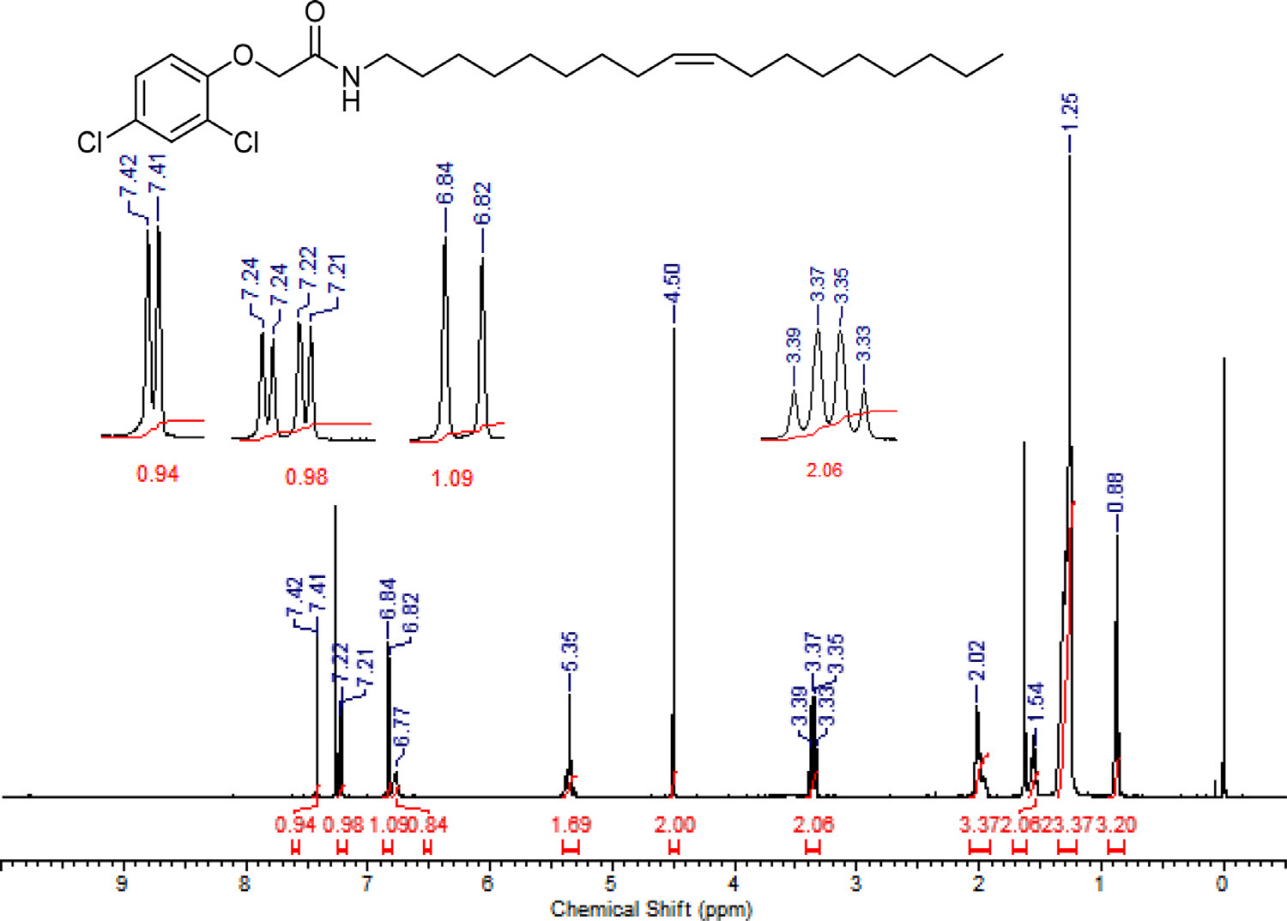
^1^H NMR (CDCl_3,_ 400 MHz) spectrum of compound **8c**.

**Fig. 14:**
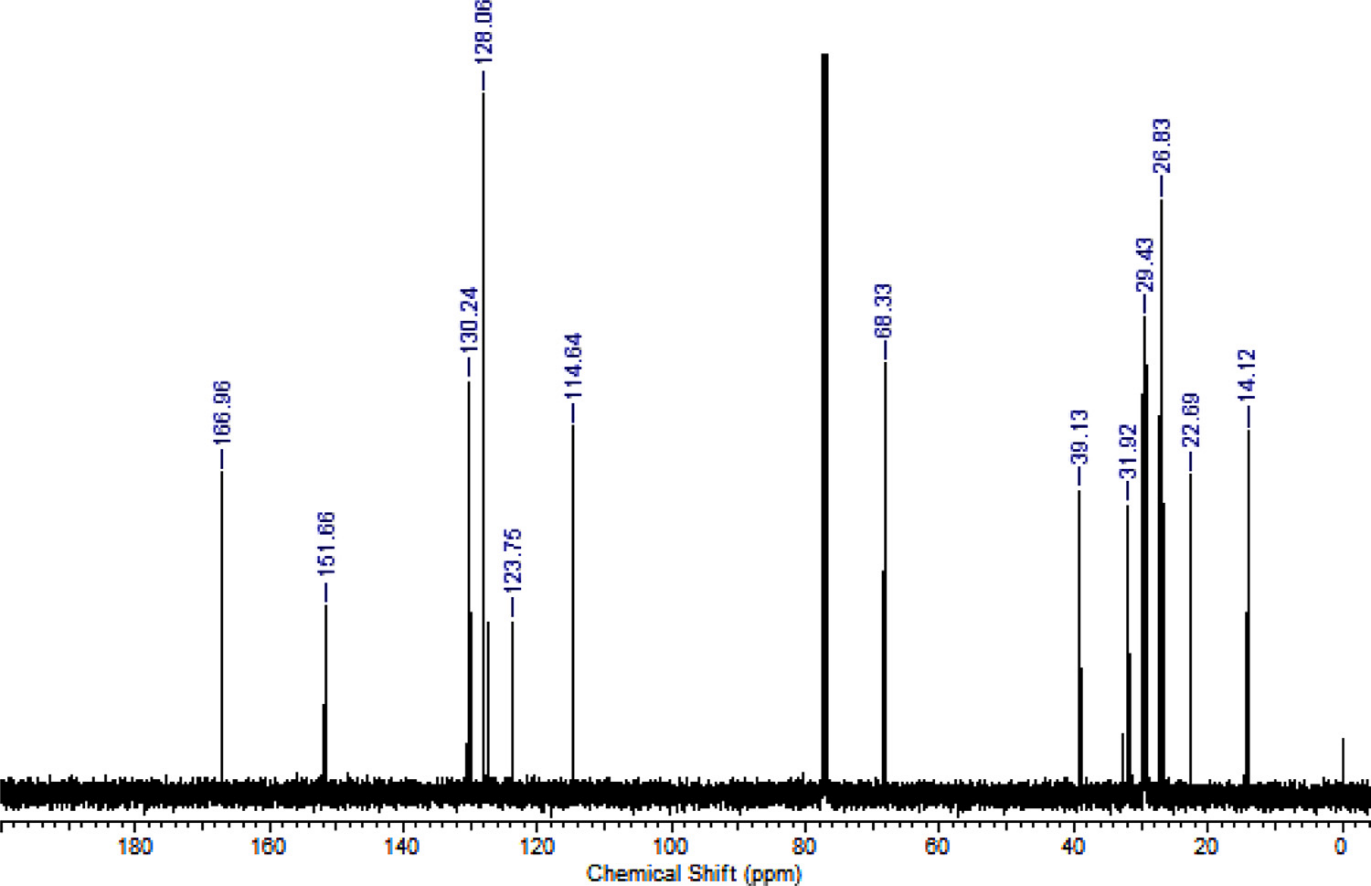
^13^C NMR (CDCl_3,_ 100 MHz) spectrum of compound **8c**.

**Fig. 15:**
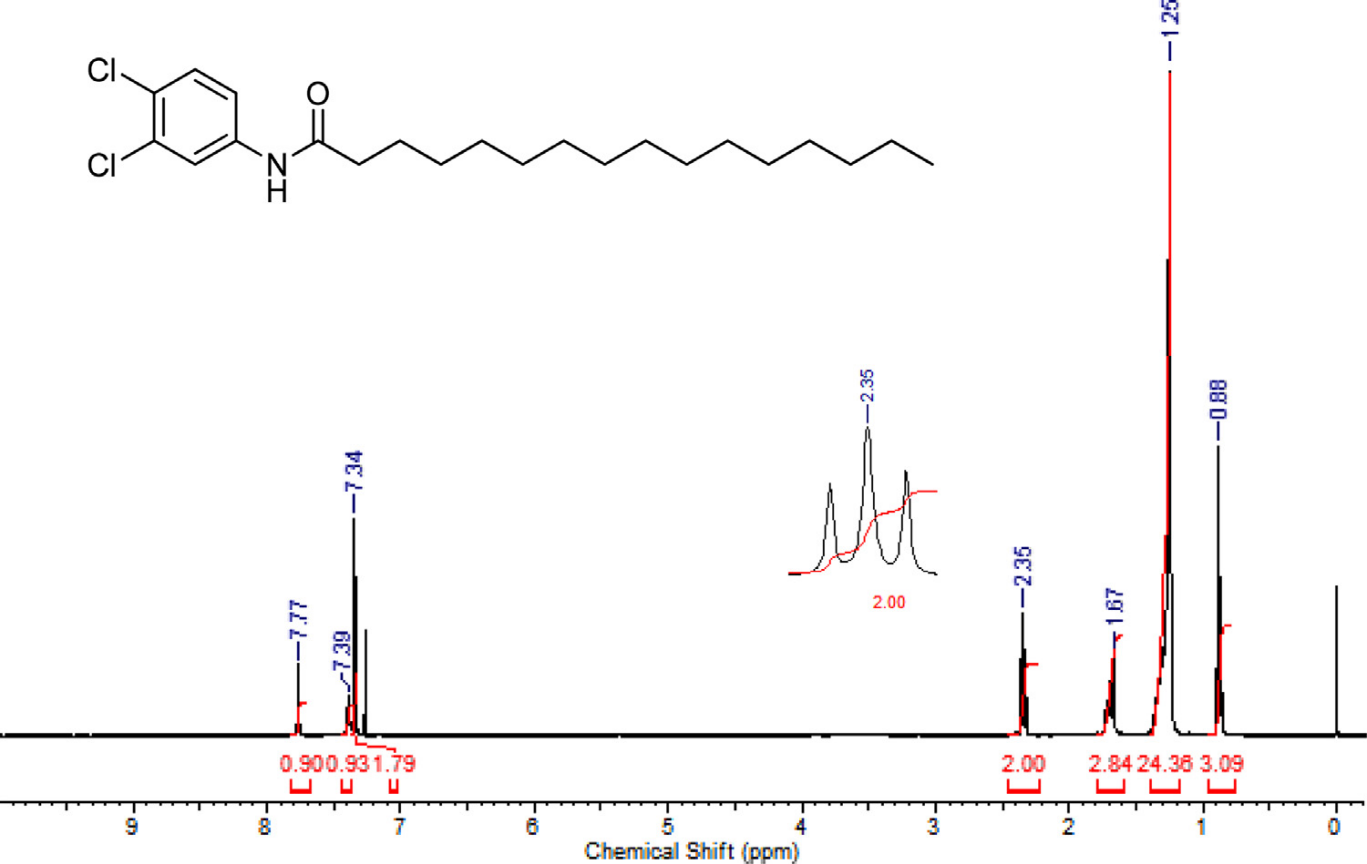
^1^H NMR (CDCl_3,_ 400 MHz) spectrum of compound **11a**.

**Fig. 16:**
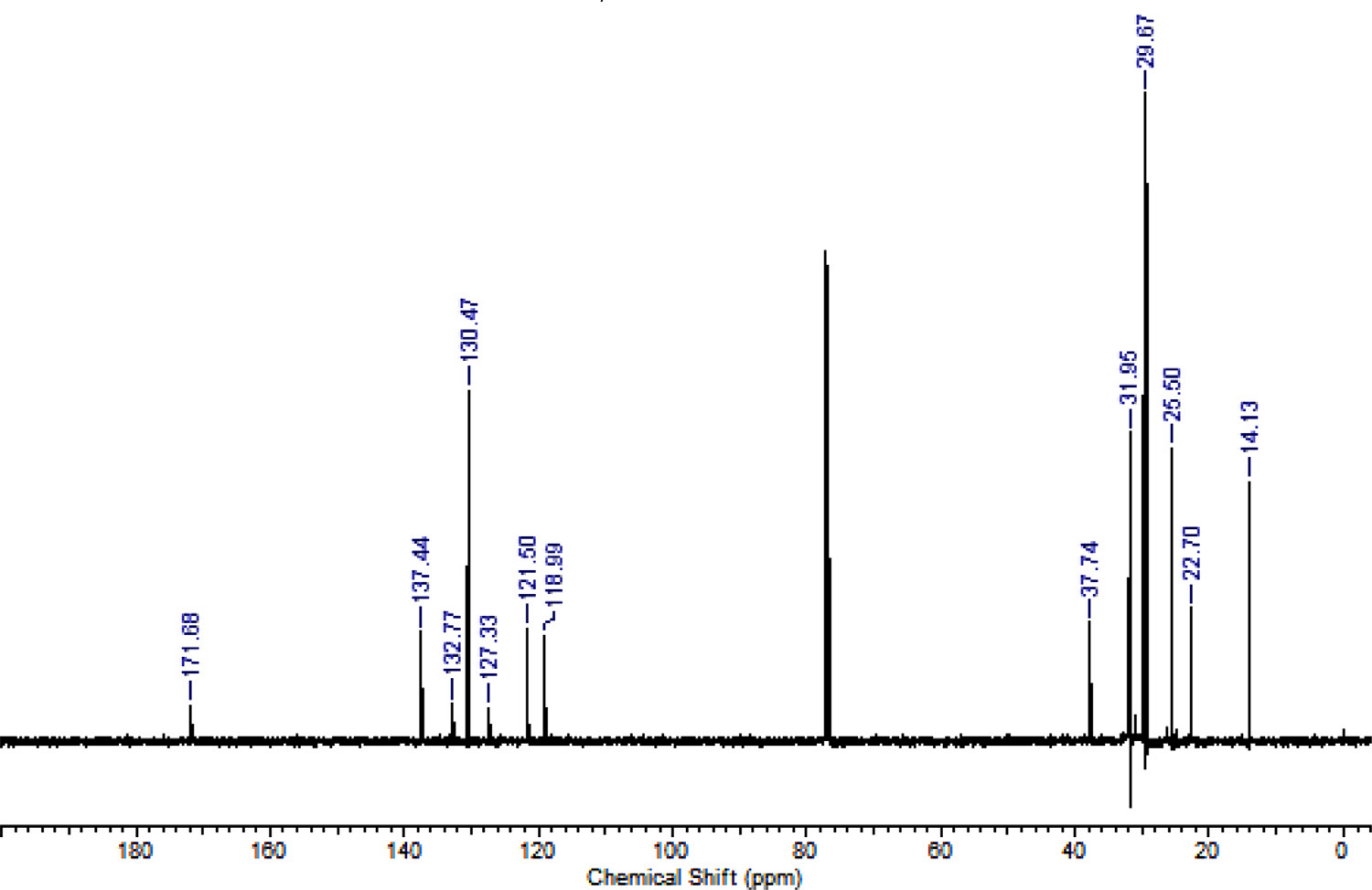
^13^C NMR (CDCl_3,_ 100 MHz) spectrum of compound **11a**.

**Fig. 17:**
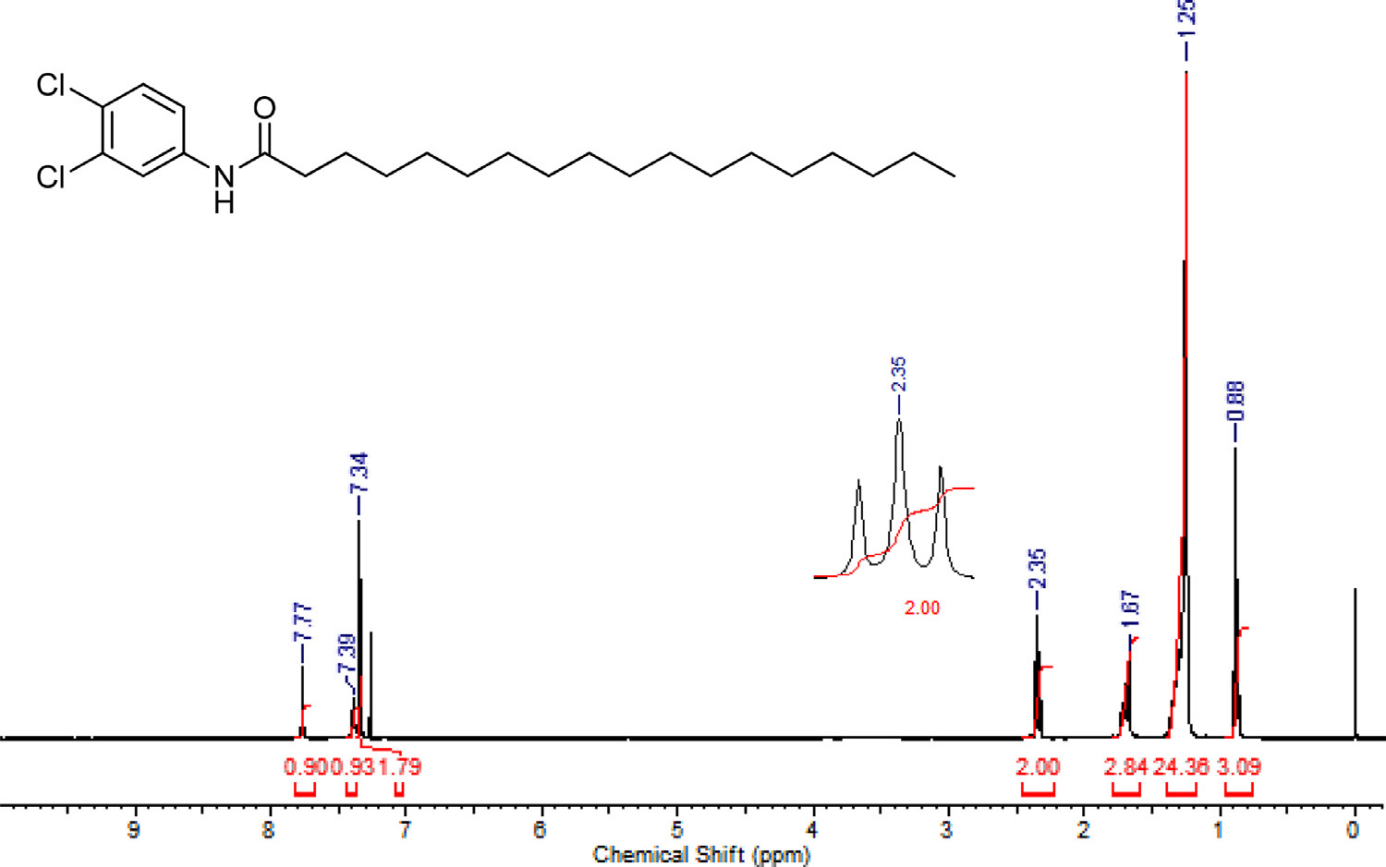
^1^H NMR (CDCl_3,_ 400 MHz) spectrum of compound **11b**.

**Fig. 18:**
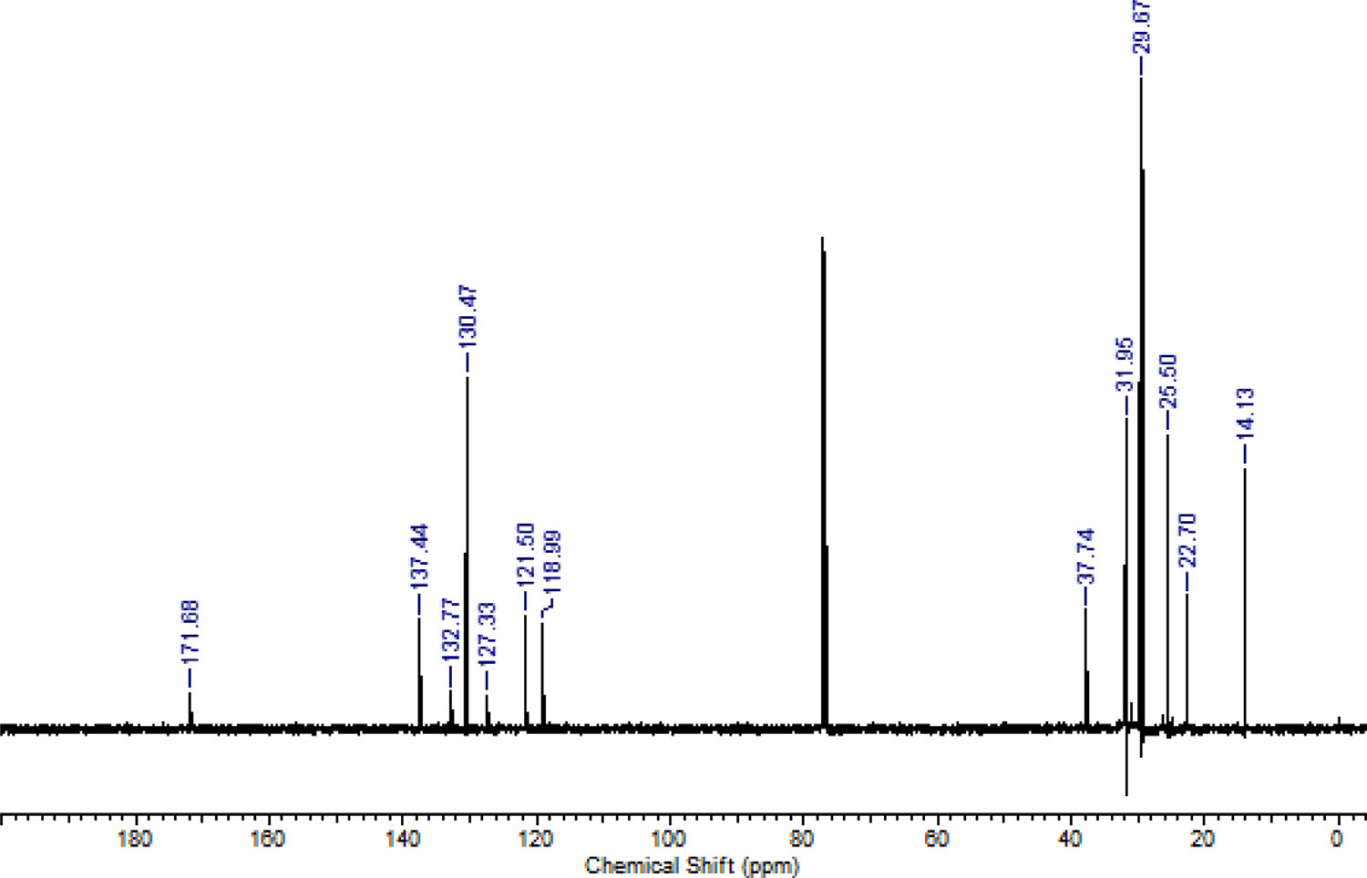
^13^C NMR (CDCl_3,_ 100 MHz) spectrum of compound **11b**.

**Fig. 19:**
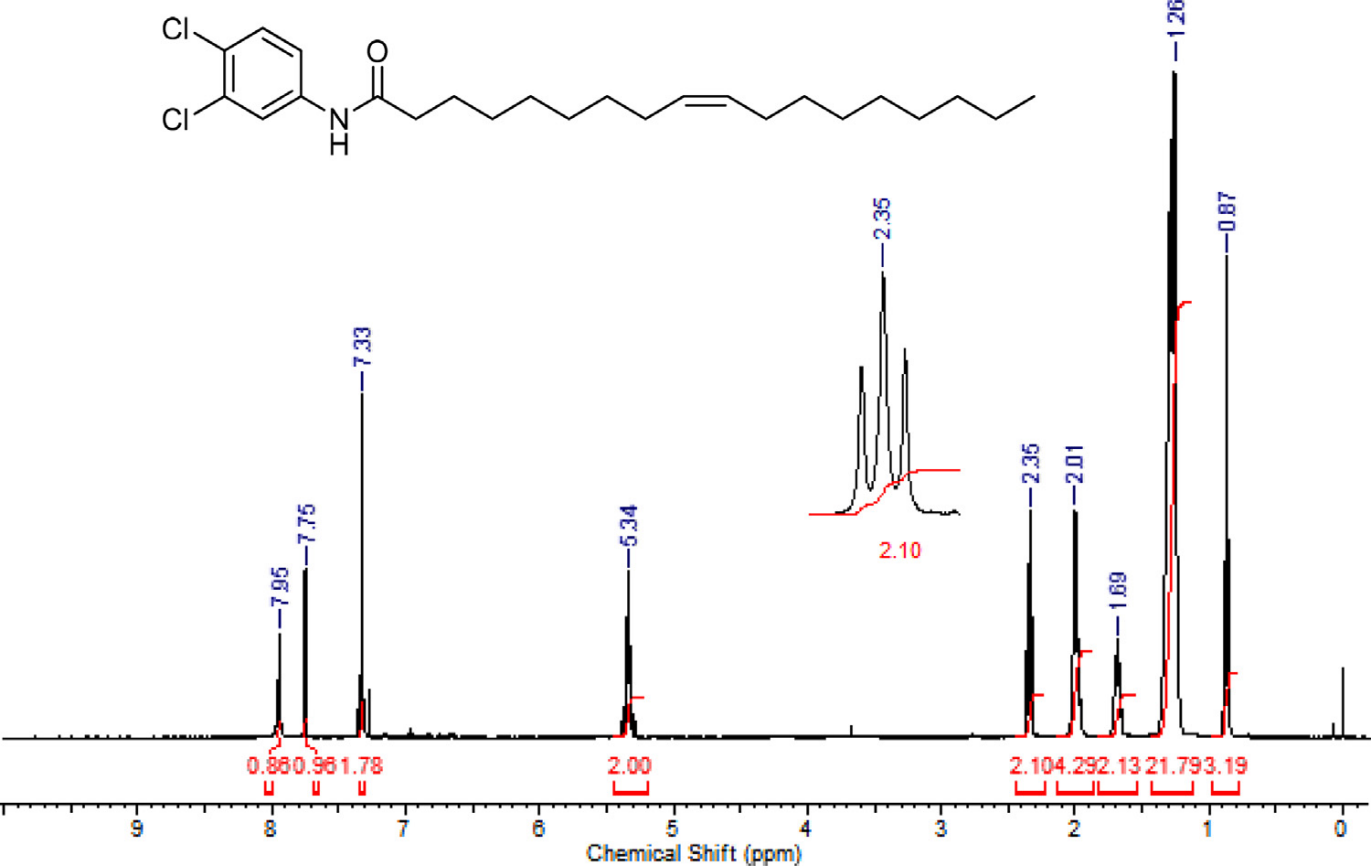
^1^H NMR (CDCl_3,_ 400 MHz) spectrum of compound **11c**.

**Fig. 20:**
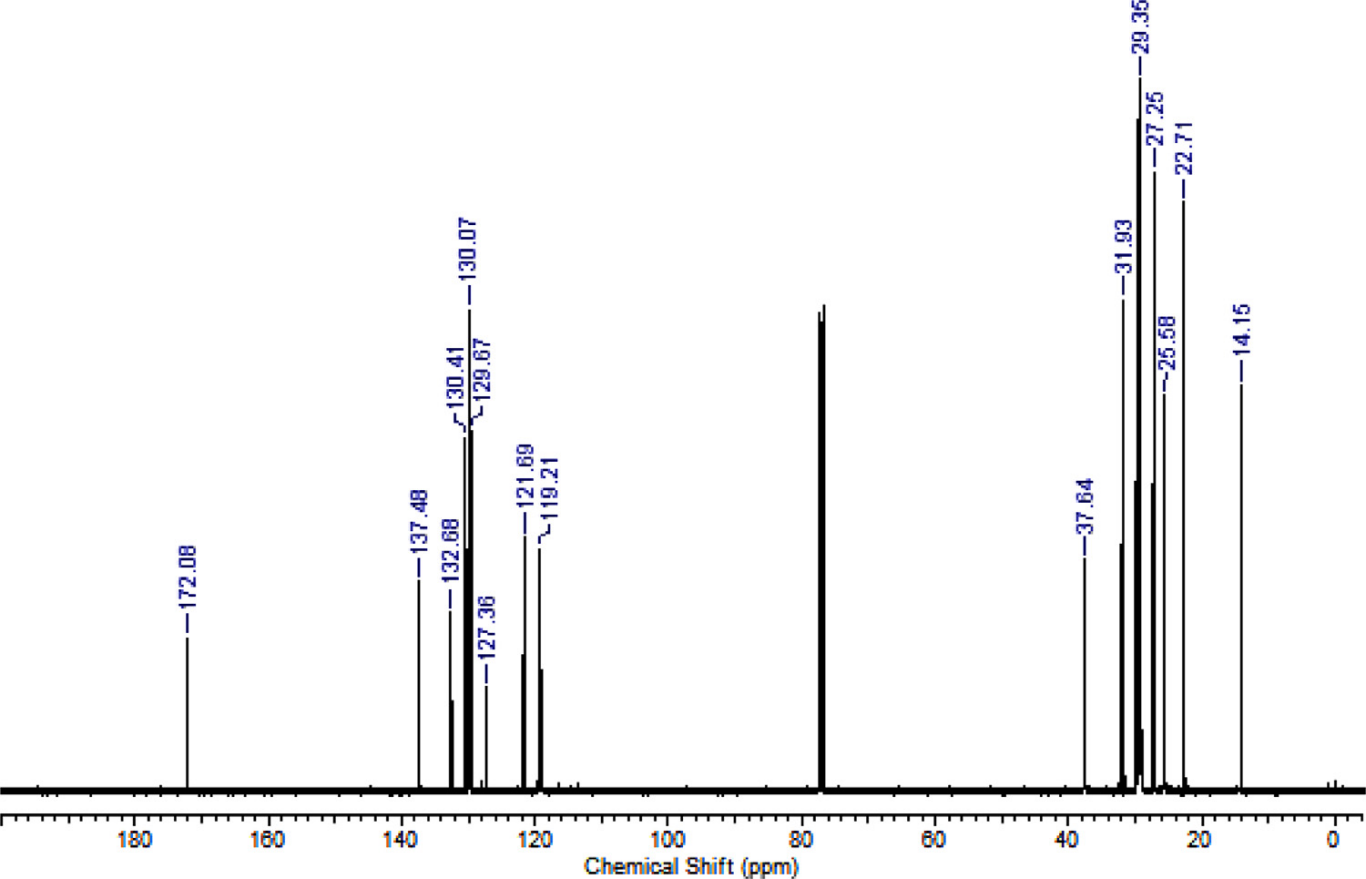
^13^C NMR (CDCl_3,_ 100 MHz) spectrum of compound **11c**.

**Fig. 21:**
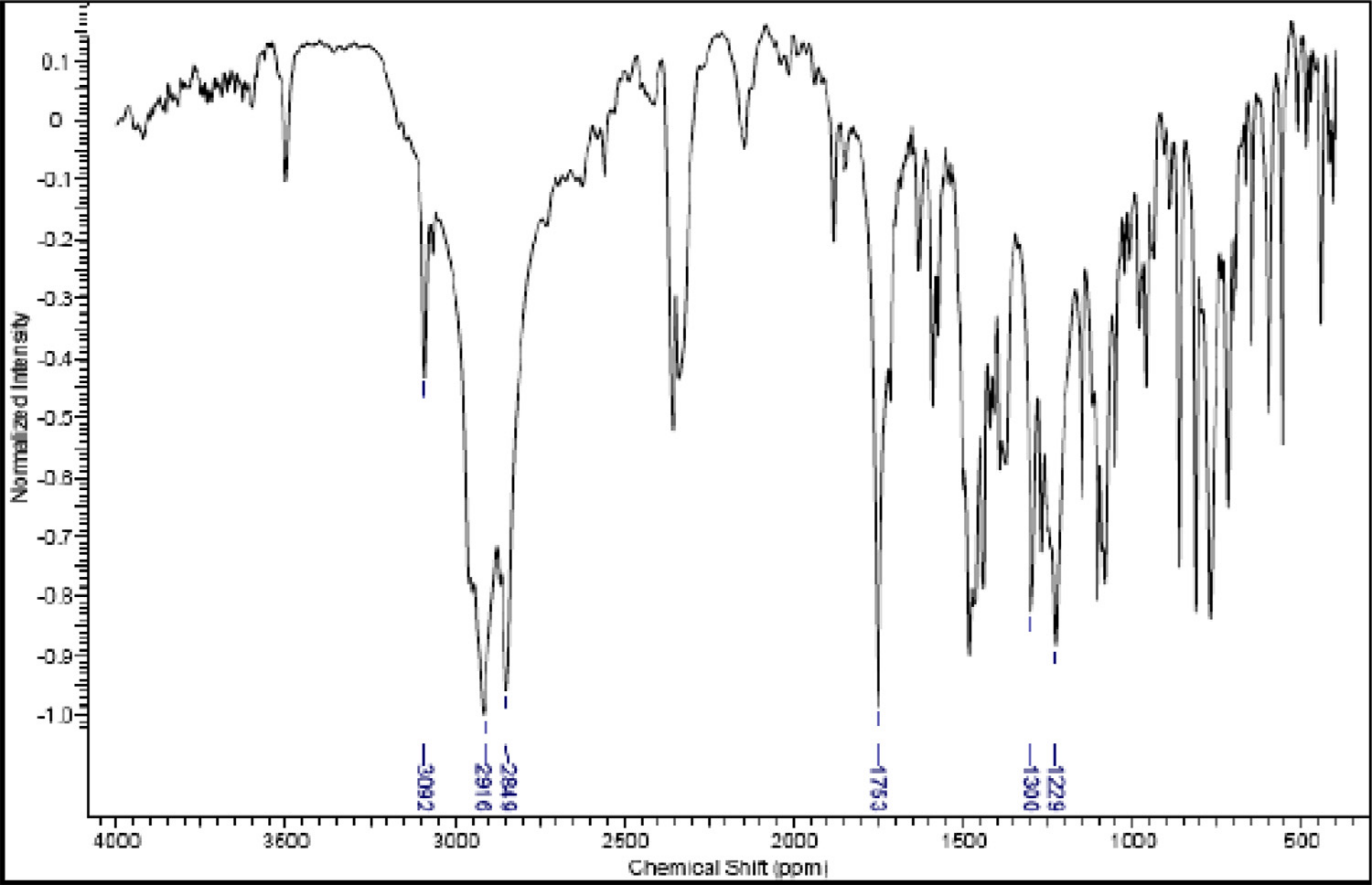
IR (cm^−1^_,_ KBr) spectrum of compound **6a**.

**Fig. 22:**
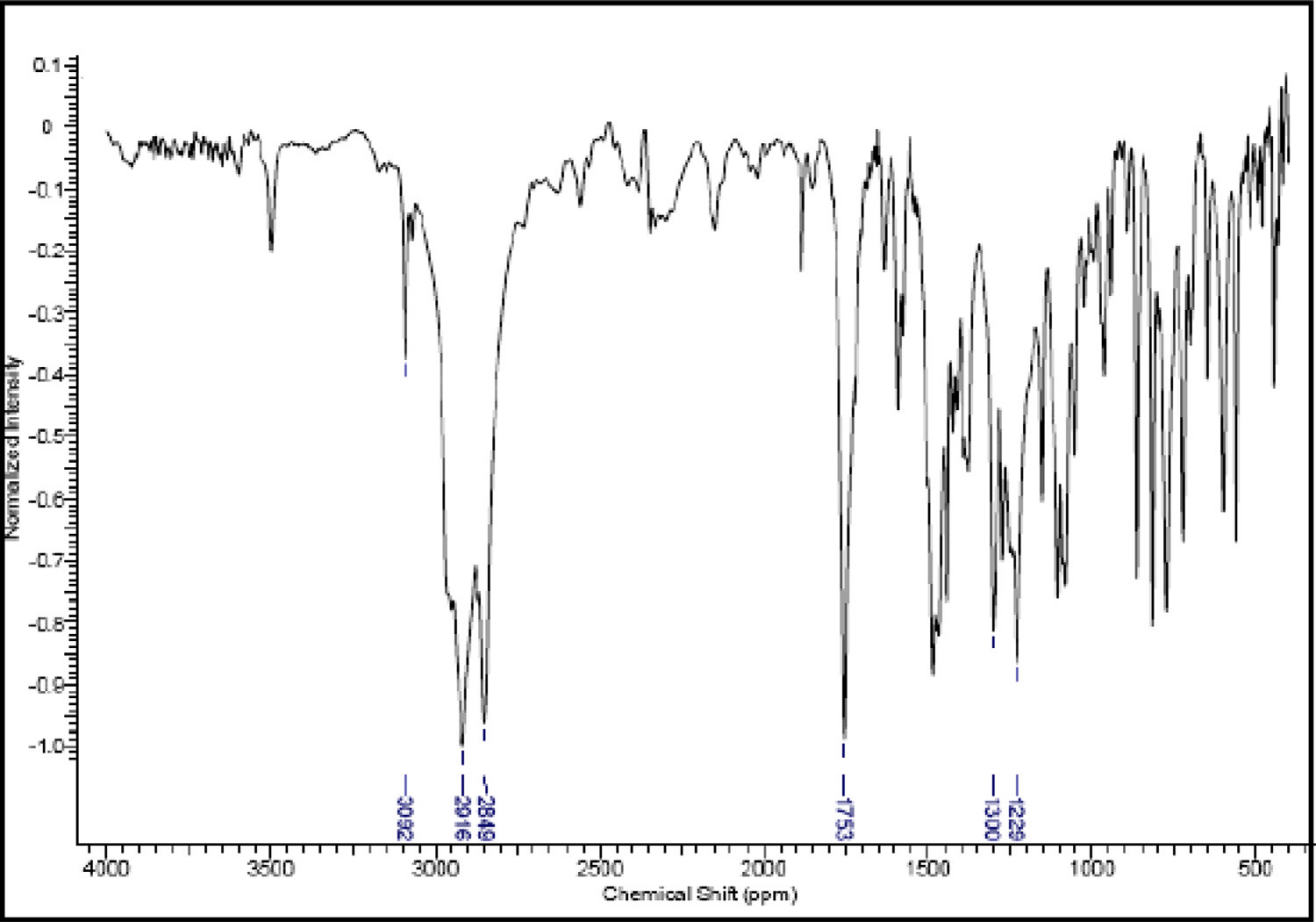
IR (cm^−1^_,_ KBr) spectrum of compound **6b**.

**Fig. 23:**
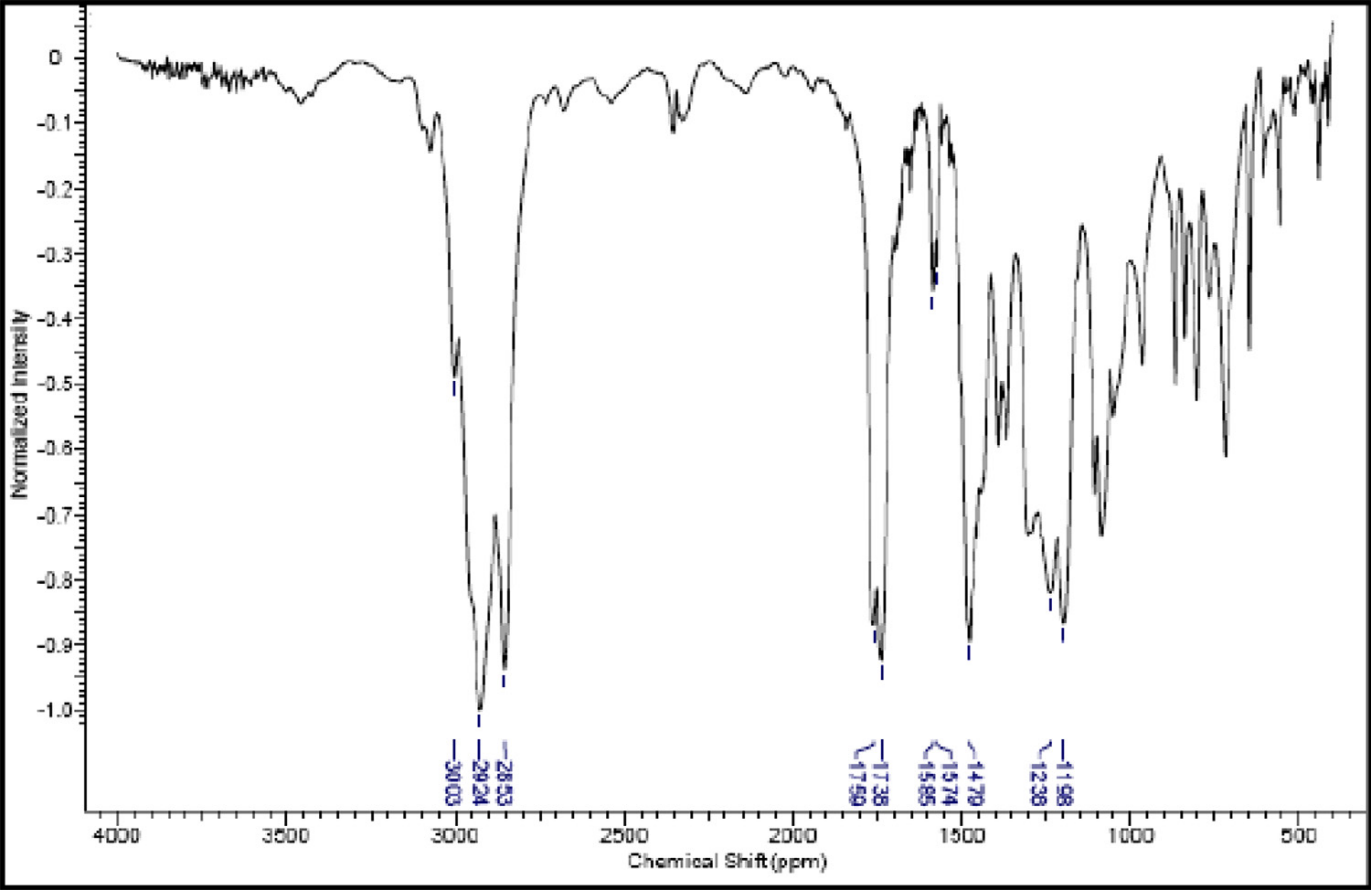
IR (cm^−1^_,_ KBr) spectrum of compound **6c**.

**Fig. 24:**
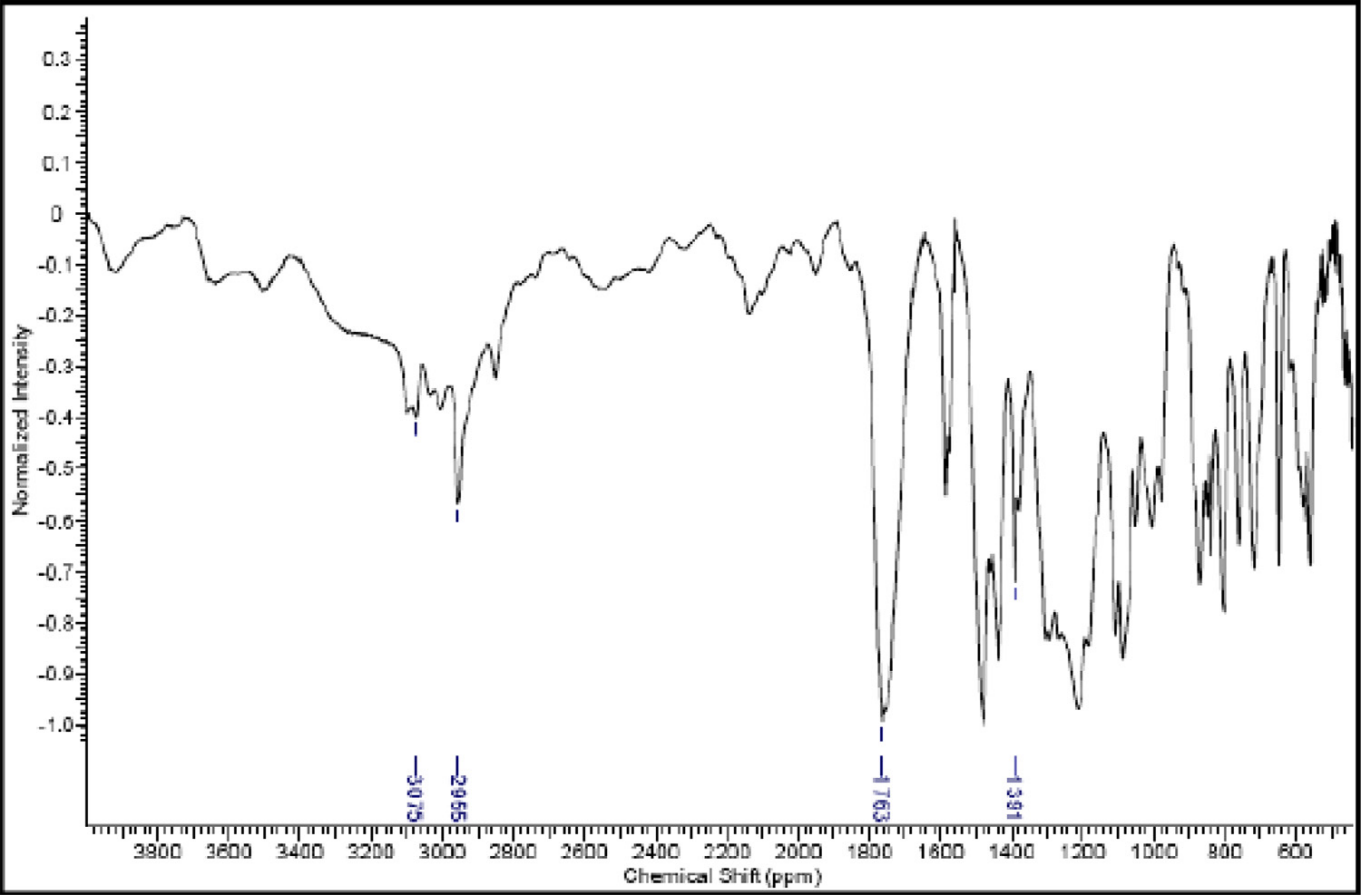
IR (cm^−1^_,_ KBr) spectrum of compound **6d**

**Fig. 25:**
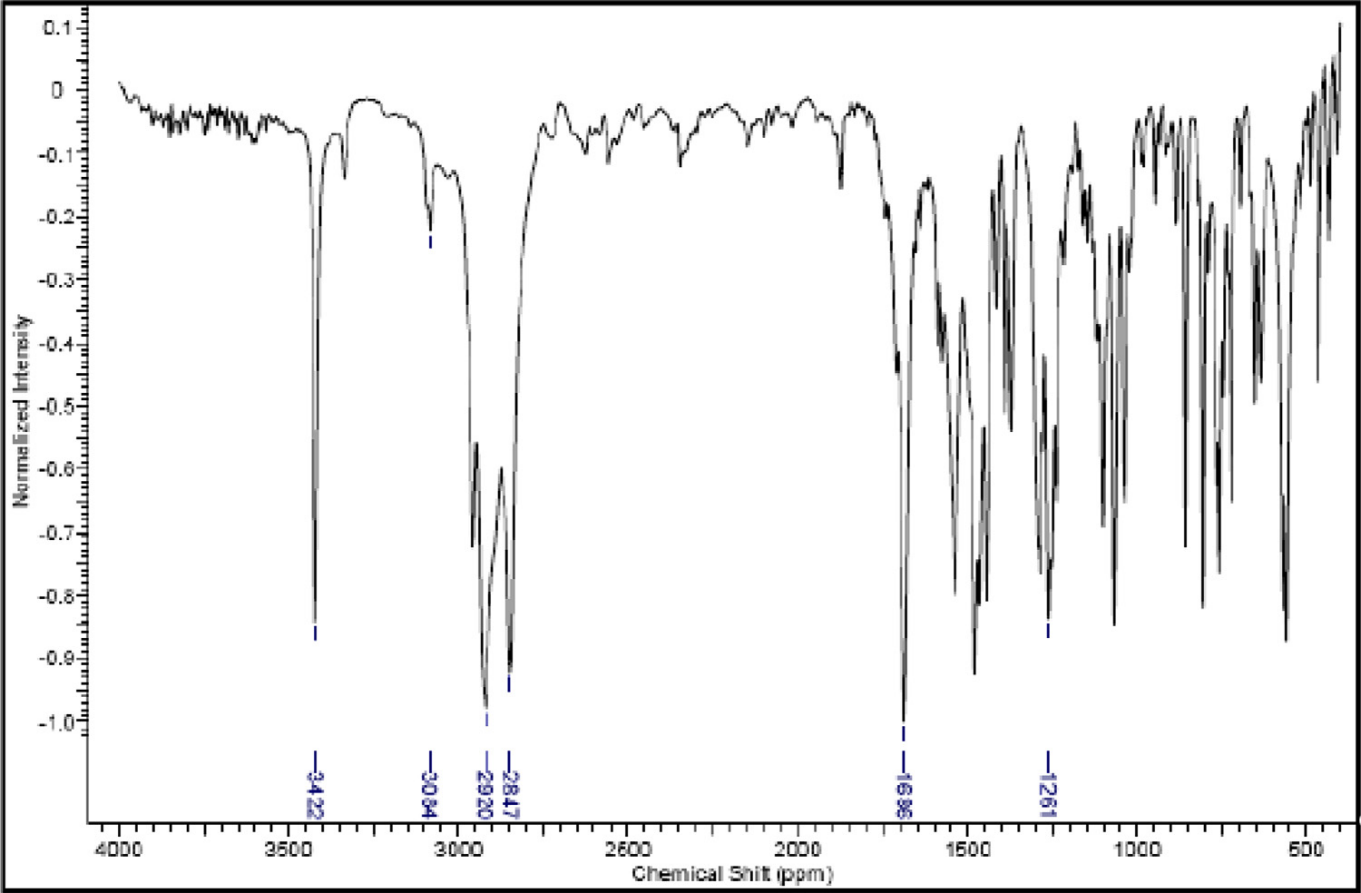
IR (cm^−1^_,_ KBr) spectrum of compound **8a**.

**Fig. 26:**
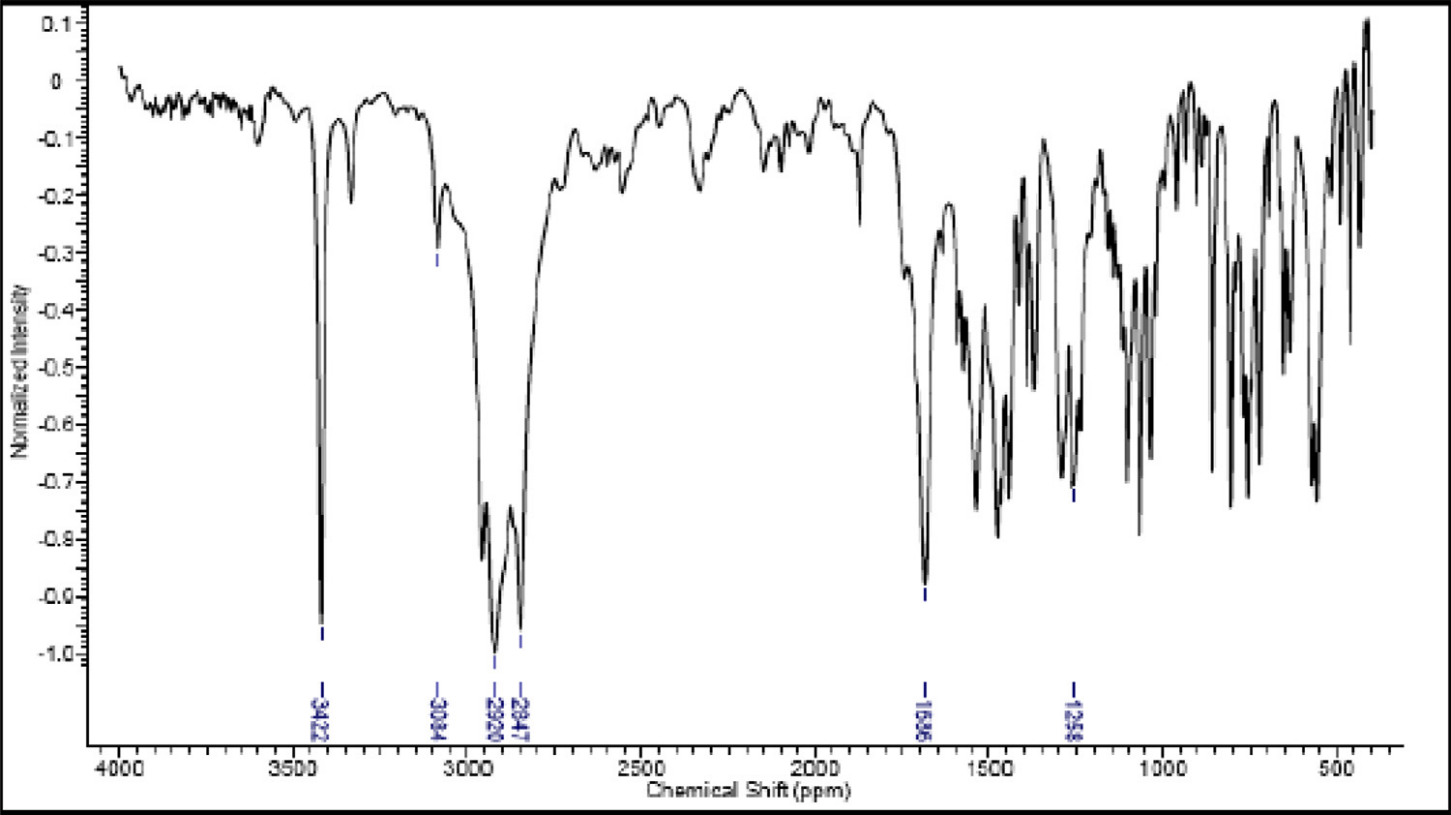
IR (cm^−1^_,_ KBr) spectrum of compound **8b**.

**Fig. 27:**
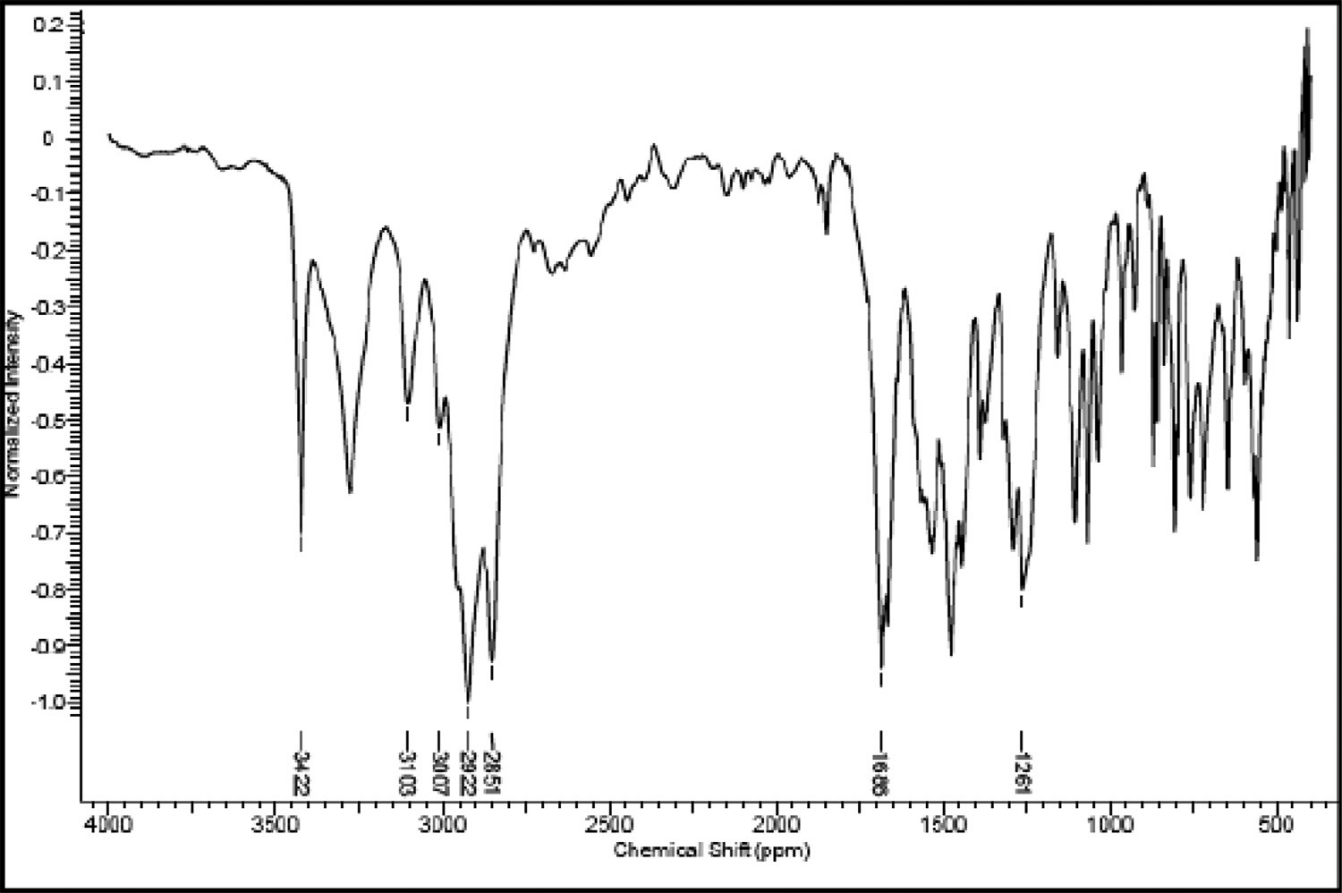
IR (cm^−1^_,_ KBr) spectrum of compound **8c**.

**Fig. 28:**
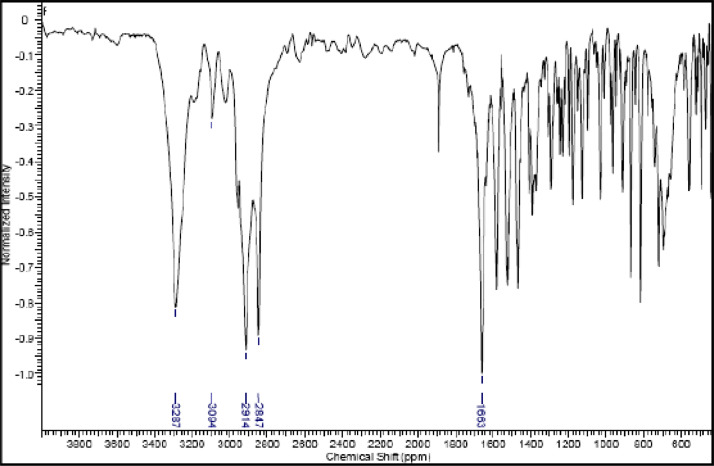
IR (cm^−1^_,_ KBr) spectrum of compound **11a**.

**Fig. 29:**
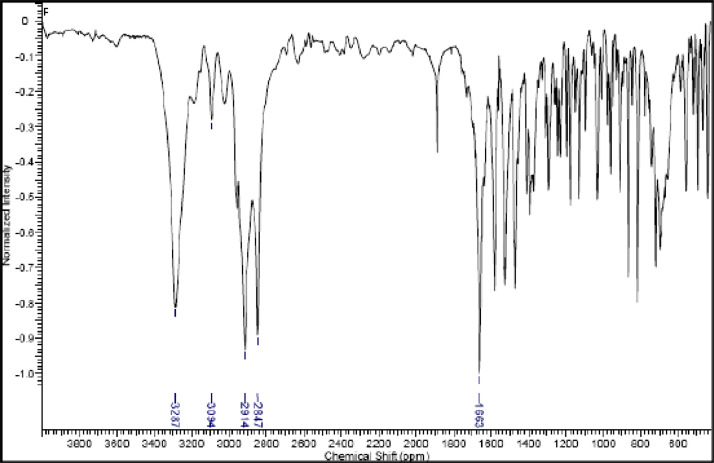
IR (cm^−1^_,_ KBr) spectrum of compound **11b**.

**Fig. 30:**
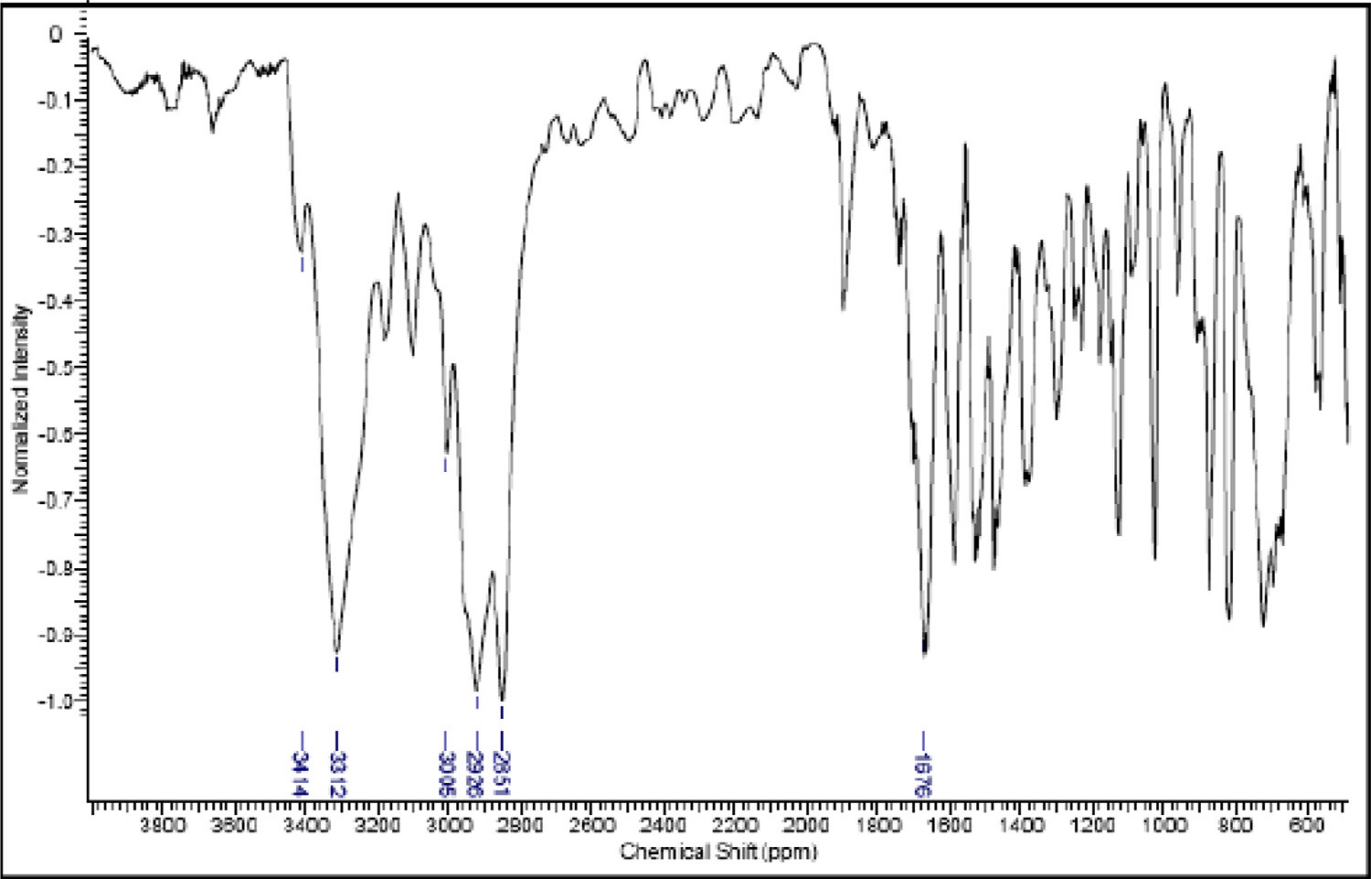
IR (cm^−1^_,_ KBr) spectrum of compound **11c**.

### 1.2 Kinetic studies by ^1^H NMR and UV–vis

**Fig. 31:**
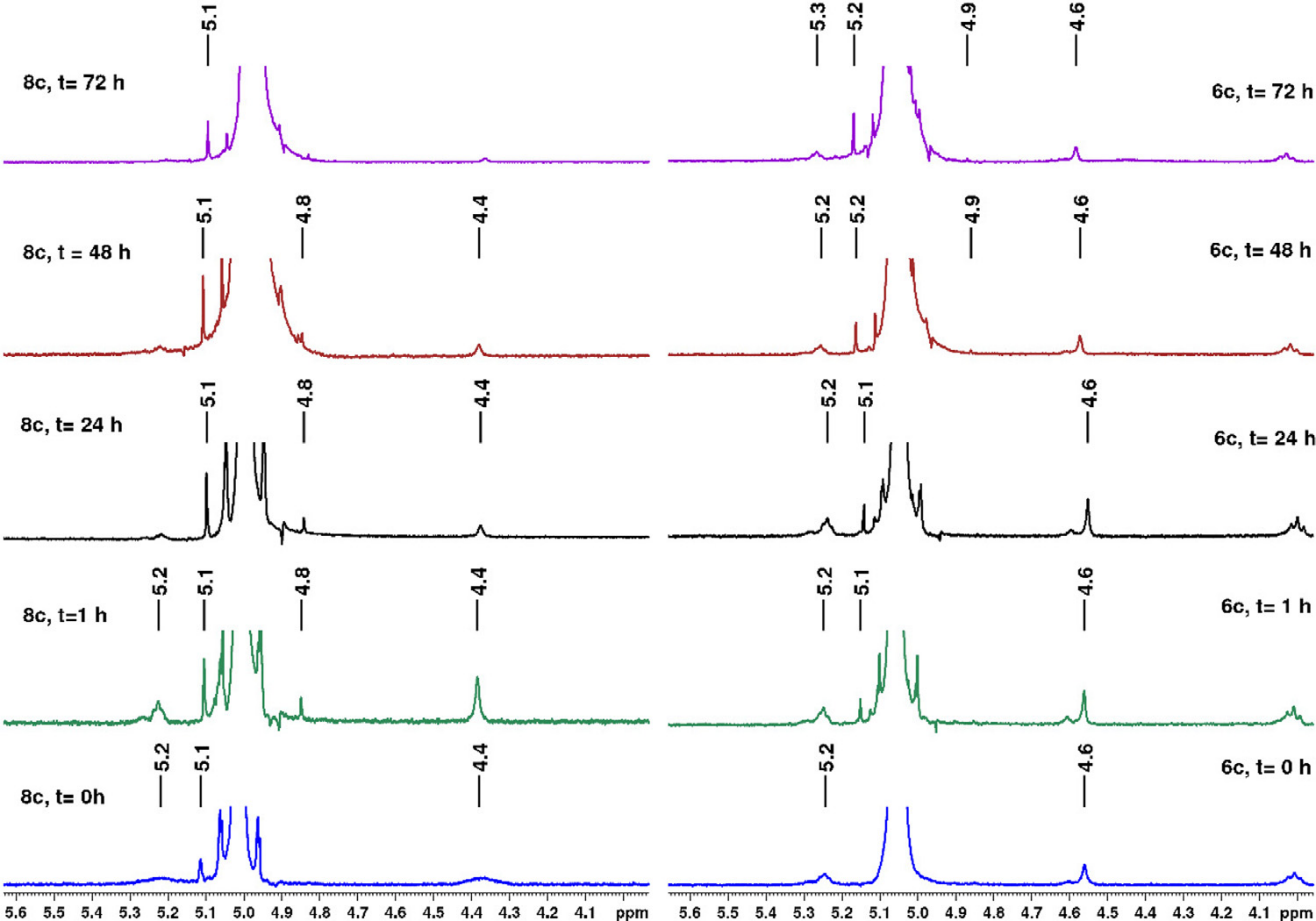
Comparation between NMR ^1^H (400 MHz, D_2_O) spectra from acid hydrolysis of fatty derivatives ester **6c** (right) and amide **8c** (left); Conditions: [**6c**]= 6.67 × 10^−5^ mol L^−1^; [**8c**]= 6.67 × 10^−5^ mol L^−1^; [HCl]=0.1 mol L^−1^; 60 °C.

**Fig. 32:**
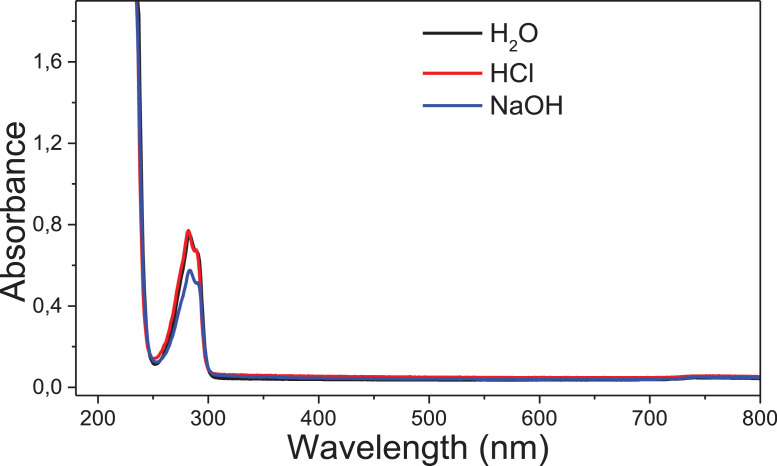
Spectra 2,4-D in neutral, acid and basic medium, showing the characteristic band of 2,4-D between 280 and 300 nm.

**Fig. 33:**
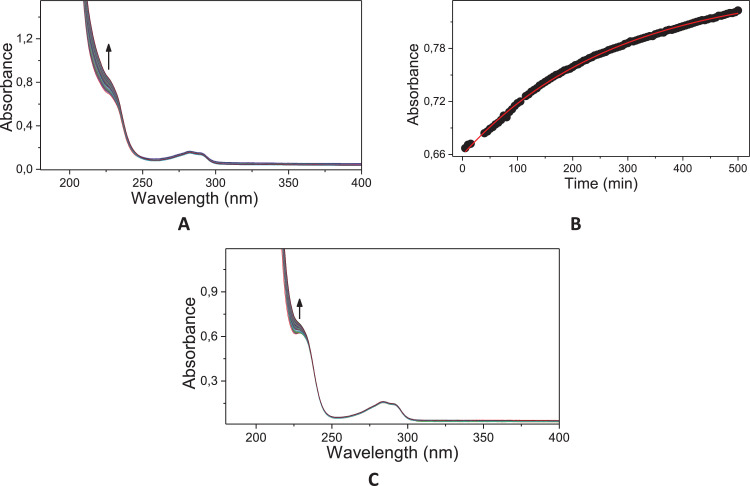
Typical consecutive spectra for the acid (A) and basic hydrolysis (C) of 2,4-D, and corresponding kinetic profile at 300 nm for the acid hydrolysis (**B**); The basic hydrolysis was too slow to be followed. Solid line corresponds to the fits according to a pseudo-first order equation.^1^ Conditions: [2,4-D]= 6.67 × 10^−5^ mol L^−1^; [HCl]=0.1 mol L^−1^; 60 °C.

**Fig. 34:**
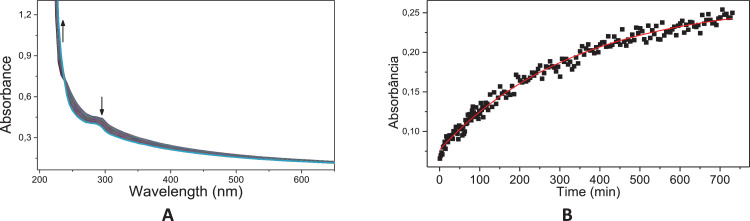
Typical consecutive spectra for the basic hydrolysis of **6c** (A); Kinetic profile at 230 nm for the basic hydrolysis of **6c** (B); Solid line corresponds to the fits according to a pseudo-first order equation; Condition: 60 °C, [6c]= 6.67 × 10^−5^ mol L^−1^; [NaOH]=0.1 mol L^−1^.

**Fig. 35:**
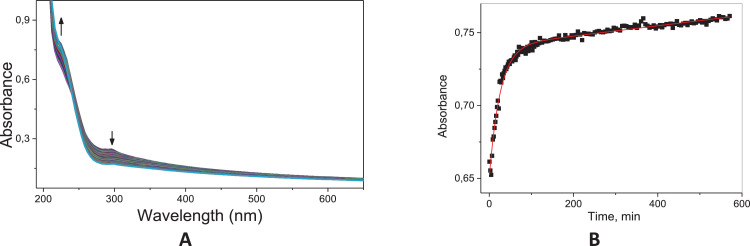
Typical consecutive spectra for the acid hydrolysis of **6c** (A); Kinetic profile at 230 nm for the acid hydrolysis of **6c** (B); Solid line corresponds to the fits according to Eq. (1); Condition: 60 °C, [6c]= 6.67 × 10^−5^ mol L^−1^; [HCl]=0.1 mol L^−1^.

**Fig. 36:**
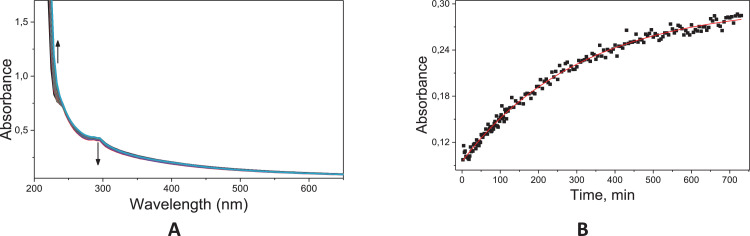
Typical consecutive spectra for the basic hydrolysis of **8c** (A); Kinetic profile at 230 nm for the basic hydrolysis of **8c** (B); Solid line corresponds to the fits according to a pseudo-first order equation; Condition: 60 °C, [8c]= 6.67 × 10^−5^ mol L^−1^; [NaOH]=0.1 mol L^−1^.

**Fig. 37:**
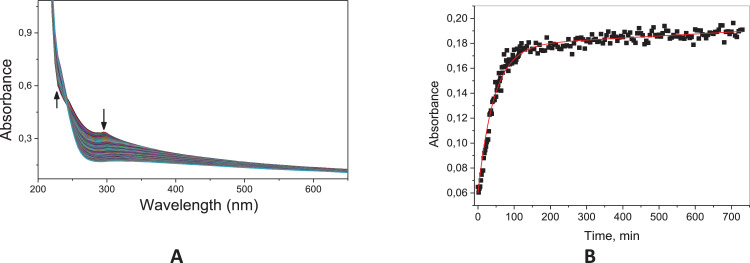
Typical consecutive spectra for the acid hydrolysis of **8c** (A); Kinetic profile at 230 nm for the acid hydrolysis of **8c** (B); Solid line corresponds to the fits according to Eq. (1). Condition: 60 °C, [**8c**]= 6.67 × 10^−5^ mol L^−1^; [HCl]=0.1 mol L^−1^.

**Fig. 38:**
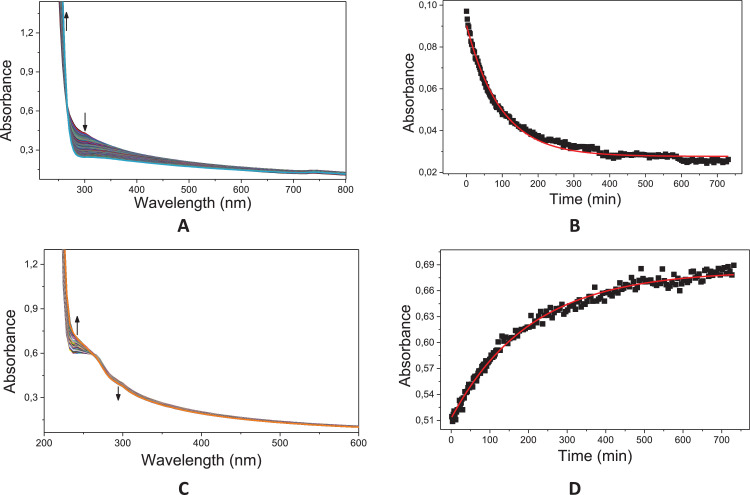
Typical consecutive spectra for the acid (A) and basic hydrolysis (C) of **11c,** and corresponding kinetic profile at 300 nm for the acid hydrolysis (**B**) and at 230 nm for the basic hydrolysis (**D**); Solid line corresponds to the fits according to a pseudo-first order equation.^1^ Conditions: [**11c**]= 6.67 × 10^−5^ mol L^−1^; [HCl]=0.1 mol L^−1^; 60 °C.

The lipophilic herbicides and 2,4-D were submitted to studies of kinetic behavior to determine the degradation's profile in aqueous medium, under basic and acid conditions. The degradation's profile studied by kinetic ^1^H NMR and UV–vis are showed in Fig. 31, Fig. 32, Fig. 33, Fig. 34, Fig. 35, Fig. 36, Fig. 37, Fig. 38.

## 2 Experimental design, materials and methods

### 2.1 NMR characterization experiments

The NMR characterization experiments of lipophilic herbicides **6a-c, 8a-c** and **11a-c** were performed in NMR 5 mm tube on a Bruker AVANCE 400 NMR spectrometer operating at 9.4T, observing ^1^H and ^13^C at 400.13 MHz and 100.50 MHz, respectively, equipped with a 5 mm direct detection probe (BBO) with gradient along the *z-*axis in CDCl_3_ or DMSO-*d6* solution with TMS as the internal standard. Fig. 21, Fig. 22, Fig. 23, Fig. 24, Fig. 25, Fig. 26, Fig. 27, Fig. 28, Fig. 29, Fig. 30.

### 2.2 Kinetic studies by UV–Vis

The kinetic studies were carried by UV–Vis spectroscopy (Agilent Cary) monitoring in the region of 190–800 nm under pseudo-first order conditions [2]. An aliquot of 20 mL stock solution of the target compounds (**6c, 8c** and **11c**; 0.01 mol.L^−1^ in acetonitrile) was added to a quartz cuvette (10 mm optical path) containing 3 mL of the reaction medium: acid solution (HCl 0.1 mol.L^−1^ – acid hydrolysis) or basic solution (NaOH 0.1 mol.L^−1^ – alkaline hydrolysis). The reactions were monitored for at least five half-life times, by following the reactant consumption and product formation. The kinetic profiles (absorbance vs time) were fitted with equations, using iterative least-squares software.

### 2.3 Kinetic studies by RMN

The experiments were performed in NMR 5 mm tube using aliquot of 20 mL stock solution of the target compounds (**6c, 8c** and **11c**; 0.01 mol.L^−1^ in acetonitrile) containing 3 mL of the reaction medium: acid solution (HCl 0.1 mol.L^−1^ – acid hydrolysis) or basic solution (NaOH 0.1 mol.L^−1^ – alkaline hydrolysis).

For qNMR ^1^H experiments, pulse was calculated by *pulsecal*. The relaxation delay for use in the acquisition of the quantitative ^1^H NMR spectra was determined by T1 measurements with the aid of the pulse sequence inversion recovery, with same parameters as for ^1^H spectra changing the τ values from 0.01 to 15 s. ^1^H spectra were acquired by using a 30° pulse sequence (*zg*) with the following parameters: 30 s of relaxation delay (D1), 16 transients, a spectral width (SW) of 4789.27 Hz (∼ 12.0 ppm), 64 K numbers of data (TD), and 6.84 s of acquisition time (AQ). The experiments were performed at 298 K. FIDs were Fourier transformed with line broadening (LB) = 0.3 Hz. The resulting spectra were manually phased and baseline corrected, and referenced to the TMS at δ 0.0 ppm.

## Declaration of Competing Interest

The authors declare that they have no known competing financial interests or personal relationships that could have appeared to influence the work reported in this paper.

## Appendix Supplementary materials

Image, application 1

Image, application 2

Image, application 3

Image, application 4

Image, application 5

Image, application 6
